# Regioselective Glycosylation of Fluorine-18-Labeled Sorbitol for Enhanced Bacterial Detection In Vivo Using PET

**DOI:** 10.1021/jacsau.5c01153

**Published:** 2025-12-01

**Authors:** Sang Hee Lee, Jung Min Kim, Marina López-Álvarez, Alexandre M. Sorlin, Mohammad Yaqoob Bhat, Joseph Blecha, Robert R. Flavell, Youngho Seo, Joanne Engel, Michael Ohliger, David M. Wilson

**Affiliations:** † Department of Radiology and Biomedical Imaging, 8785 University of California, San Francisco, San Francisco, California 94158, United States; ‡ Department of Medicine, University of California, San Francisco, San Francisco, California 94158, United States; § Department of Radiology, Zuckerberg San Francisco General Hospital, San Francisco, California 94110, United States

**Keywords:** ^18^F-sugar alcohol, bacteria, infection imaging, positron emission tomography, glycosylation

## Abstract

Precise and rapid detection of bacterial infection in vivo remains a significant challenge in clinical practice. In response to this challenge, several pathogen-specific positron emission tomography (PET) tracers have been developed, including the fluorine-18-labeled sorbitol derivative [^18^F]­FDS, which shows great promise in detecting bacterial infections in patients. In this study, we tested the hypothesis that the diagnostic performance of [^18^F]­FDS could be modulated via regioselective glycosylation to improve radiotracer stability, broaden organism sensitivity, and tune pharmacodynamics. A synthetic sequence was developed, whereby the common radiotracer [^18^F]­FDG was converted chemoenzymatically to α- and β-linked disaccharides via reverse phosphorolysis and subsequently reduced to the corresponding glycosylated [^18^F]­FDS derivatives. This strategy allowed the syntheses of glucopyranosyl-d-sorbitol analogs [^18^F]­FNT (α-1,3 linked), [^18^F]­FMT (α-1,4 linked), [^18^F]­FLT (β-1,3 linked), and [^18^F]­FCT (β-1,4 linked). Among these tracers, the α-linked analogs [^18^F]­FNT and [^18^F]­FMT showed greater uptake in both Gram-positive and Gram-negative pathogens compared to the β-linked analogs [^18^F]­FLT and [^18^F]­FCT. In vivo time–course PET imaging of [^18^F]­FNT and [^18^F]­FMT in uninfected mice revealed favorable pharmacokinetics, including rapid urinary excretion, minimal hepatobiliary retention, and low off-target signals. PET imaging using [^18^F]­FNT and [^18^F]­FMT detected *Klebsiella pneumoniae* pulmonary infections in mice with high infected/uninfected tissue ratios (∼6-fold). [^18^F]­FNT also showed high infected/uninfected tissue ratios (∼28-fold) in *Staphylococcus aureus* myositis, whereas the parent [^18^F]­FDS tracer was not taken up by the Gram-positive organisms tested. Our findings highlight the potential for PET tracer glycosylation as a tool to modulate target specificity and improve imaging sensitivity. These results also establish [^18^F]­FNT as a highly promising PET tracer with a high translational potential for detecting bacterial infection in vivo.

## Graphical abstract

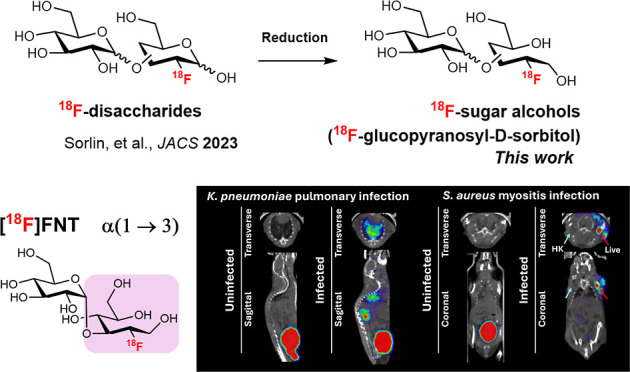

## Introduction

Sugar and sugar alcohols are versatile scaffolds for molecular imaging, especially using positron emission tomography (PET), due to their ability to interrogate conserved metabolic pathways across diverse biological systems in both mammalian cells and microorganisms.^1,2^ The glucose derivative [^18^F]­FDG is the most frequently used sugar-based PET radiotracer, used to characterize dysregulated metabolism in many oncological and inflammatory conditions. However, the clinical utility of [^18^F]­FDG in infectious diseases is limited due to its inability to distinguish between malignancy, inflammation, and infection. [^18^F]­FDG PET primarily images the host immune response, therefore potentially leading to false positive exams in conditions such as autoimmune disease or postsurgical inflammation.^3,4^ One approach to developing an infection-specific radiotracer is by targeting pathogen-specific metabolic pathways that are absent or inactive in mammalian cells, thereby ensuring high specificity and minimizing host uptake.^5^ Several recently reported PET tracers exploit metabolic differences between bacteria and humans,^6^ including [^18^F]­FDS (2-deoxy-2-[^18^F]­fluoro-d-sorbitol), a representative pathogen-targeted PET radiotracer that can be readily synthesized from [^18^F]­FDG via rapid NaBH_4_-mediated reduction.^7^ [^18^F]­FDS demonstrates excellent specificity for *Enterobacteriaceae* in vitro and in vivo without significant uptake in mammalian tissues and can rapidly differentiate infection from sterile inflammation.^8^ However, the lack of sensitivity of [^18^F]­FDS in Gram-positive pathogens such as *Staphylococcus* may limit its utility in broader clinical settings. In patients, [^18^F]­FDS accumulates in the liver and intestine, likely due to endogenous metabolic activity, potentially complicating the detection of bacteria in these locations.^9^

We hypothesized that the diagnostic performance of [^18^F]­FDS could be modified via glycosylation, potentially decreasing its mammalian background signals and improving organism sensitivity and pharmacokinetics for patient use. Glycosylation is frequently applied to drugs, especially biologics,^10,11^ but has not been previously reported for small-molecule PET tracers. Based on the radiosynthesis of [^18^F]­FDS and the reported synthesis of disaccharides via reverse phosphorolysis,^12,13^ generation of glycosylated [^18^F]­FDS and other sugar–sugar alcohols appeared feasible. A sugar–sugar alcohol is a glycoside composed of a monosaccharide and a sugar alcohol via a glycosidic linkage. A nonreducing primary sugar is unable to convert to a sugar alcohol, while a secondary sugar with a reducing end can be easily converted into a sugar alcohol in the presence of sodium borohydride.^14^ Although research on sugar–sugar alcohols is limited, maltitol (α-glucopyranosyl-(1 → 4)-d-sorbitol) has been characterized due to its natural abundance and the accessibility of its parent disaccharide (maltose, α-glucopyranosyl-(1 → 4)-d-glucose). Maltitol can be found in roasted malt and chicory leaves and is also produced from starch.^15^ Owing to its limited intestinal absorption and favorable digestive tolerance, it is widely used as a low-calorie sweetener in sugar-free foods and pharmaceuticals, although its biological functions remain unclear. A previous study revealed that maltitol is cleared quickly from the body, mostly eliminated within 1 h following intravenous administration, likely due to the absence of glucoamylase and maltase in the bloodstream and major organs except the gastrointestinal tract.^16^ In addition, maltitol exhibited a 1000-fold lower hydrolysis rate by glucoamylase compared to maltose, supporting its high metabolic stability in vivo.^17^ Consistent with its high stability and favorable pharmacokinetics, a recent study demonstrated the potential of maltitol as a tumor-selective MRI imaging agent in a 9L glioma rat model.^18^ The reported relative hydrolysis rate of nigeritol (α-glucopyranosyl-(1 → 3)-d-sorbitol) is even slower than that of maltitol (over 2000-fold slower than maltose), further supporting its high metabolic stability and low off-target retention.^17^

The glycosidic modification of [^18^F]­FDS might therefore improve the imaging contrast via rapid clearance and low hepatobiliary accumulation. Bacteria have a unique phosphoenolpyruvate-dependent phosphotransferase (PTS) system and/or ATP-binding cassette (ABC) transporters to import various carbohydrates into cells and may also selectively recognize and transport sugar–sugar alcohols such as maltitol and nigeritol.^19–21^ We therefore investigated ^18^F-glucopyranosyl-d-sorbitol analogs with both α- and β-glycosidic linkages between glucose and [^18^F]­FDS to assess whether glycosylation could improve bacterial detection in vivo. Our study demonstrated that the α-glycosylated derivatives [^18^F]­FNT and [^18^F]­FMT not only had lower off-target accumulation versus [^18^F]­FDS itself but also showed enhanced organism sensitivity, allowing the detection of Gram-positive organisms including *Staphylococcus aureus*. The α-1,3 linked tracer [^18^F]­FNT showed robust uptake by infected tissues in preclinical models of *S. aureus* myositis and *Klebsiella pneumoniae* pneumonia and is a promising tracer for patient imaging.

## Results

### Efficient Radiosyntheses of ^18^F-Labeled Glucopyranosyl-d-sorbitol Analogs via Reverse Phosphorolysis of [^18^F]­FDG Followed by Borohydride Reduction

We previously reported the chemoenzymatic syntheses of ^18^F-disaccharides from clinical [^18^F]­FDG and α/β glucose-1-phosphate via reverse phosphorolysis.^12^ By applying the general method for reduction of sugars using sodium borohydride,^14^ ^18^F-disaccharides ([^18^F]­FSK, [^18^F]­FDM, [^18^F]­FDL, and [^18^F]­FDC) were quantitatively converted into the corresponding ^18^F-glucopyranosyl-d-sorbitols ([^18^F]­FNT, [^18^F]­FMT, [^18^F]­FLT, and [^18^F]­FCT) in 20 min (*n* ≥ 3), confirmed by radio-TLC (Figures 1A and S1). These four ^18^F-glucopyranosyl-d-sorbitol analogs differ in the glycosidic linkage position and stereochemistry: [^18^F]­FMT and [^18^F]­FNT (2-deoxy-2-[^18^F]­fluoro-d-maltitol/nigeritol) are linked via α-linkage at 1 → 4 and 1 → 3, respectively, while [^18^F]­FCT and [^18^F]­FLT (2-deoxy-2-[^18^F]­fluoro-d-cellobiotol/laminaribiotol) are linked via β-linkage at 1 → 4 and 1 → 3, respectively (Figure 1B). [^18^F]­FDS was synthesized according to the published method.^22^

**Figure 1:**
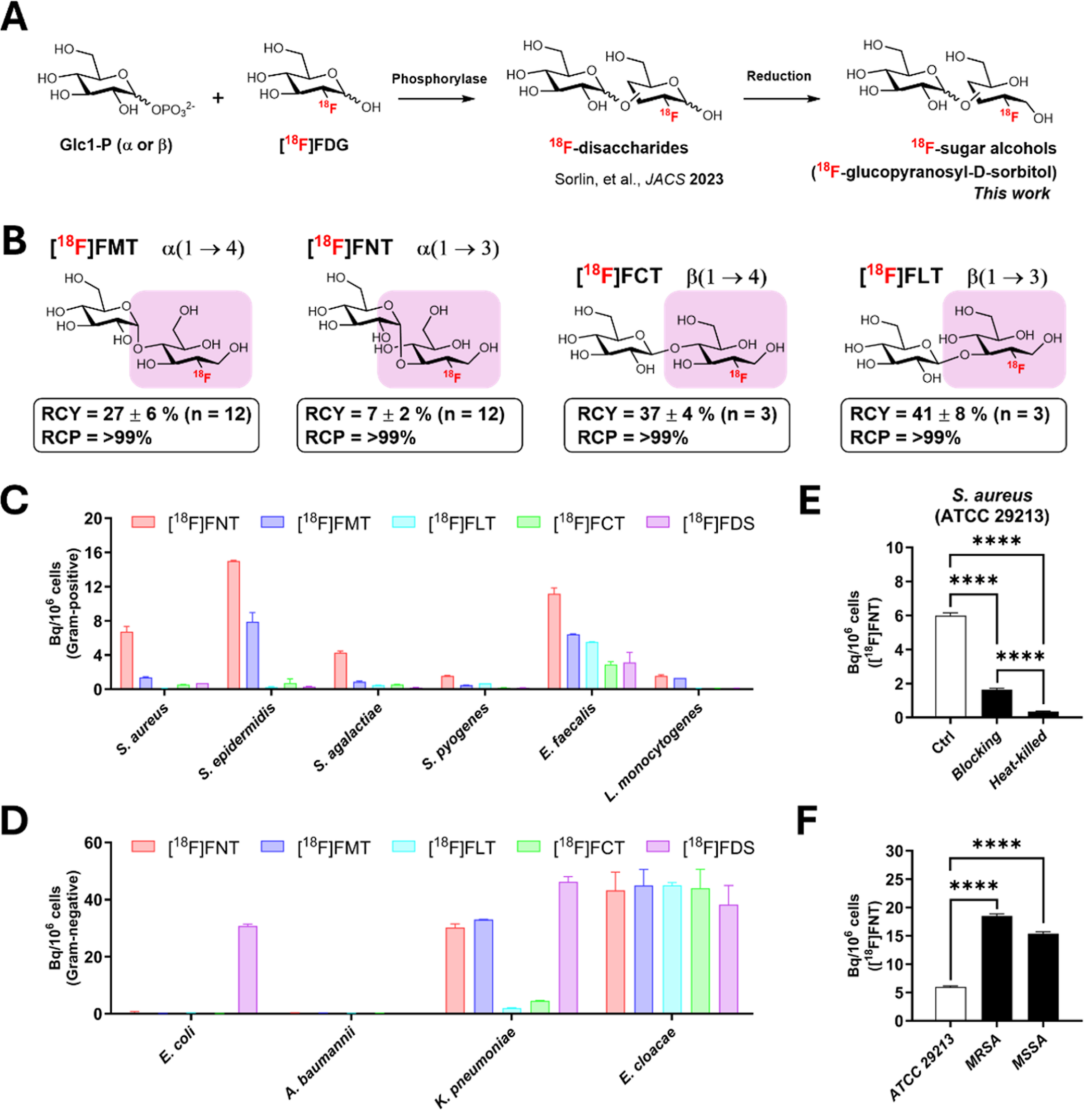
Synthesis and in vitro evaluation of ^18^F-glucopyranosyl-d-sorbitol analogs. (A) Schematic of the synthesis of ^18^F-glucopyranosyl-d-sorbitol analogs from [^18^F]­FDG via reverse phosphorolysis and subsequent reduction. (B) Chemical structures of ^18^F-glucopyranosyl-d-sorbitol analogs used in this study, which differ by glycosidic linkages and stereochemistry. The highlighted portions of analogs correspond to the [^18^F]­FDS scaffold. RCY: radiochemical yield (nondecay corrected), RCP: radiochemical purity. (C,D) In vitro uptake of [^18^F]­FNT, [^18^F]­FMT, [^18^F]­FLT, [^18^F]­FCT, and [^18^F]­FDS (control) by the indicated Gram-positive (C) and Gram-negative (D) bacterial pathogens. (E) Specificity of [^18^F]­FNT uptake in *S. aureus* (Ctrl, ATCC 29213) reflected by reduced uptake in the presence of [^19^F]­FNT (1 mM, blocking) and in heat killed bacteria. (F) Differential uptake of [^18^F]­FNT in methicillin-resistant *S. aureus* (MRSA) and methicillin-sensitive *S. aureus* (MSSA) clinical isolates compared to reference strain (*S. aureus* ATCC 29213; MSSA). ****P < 0.0001 by unpaired Student’s *t*-test.

### α-Linked Glycosylated [^18^F]­FDS Analogs [^18^F]­FNT and [^18^F]­FMT Accumulate in Several Pathogenic Bacteria In Vitro, Including *S. aureus*

Given their potential for bacterial accumulation, we screened four ^18^F-glucopyranosyl-d-sorbitols, [^18^F]­FNT, [^18^F]­FMT, [^18^F]­FLT, and [^18^F]­FCT, in a panel of clinically relevant Gram-positive and Gram-negative bacterial pathogens. [^18^F]­FNT showed moderate uptake in *S. aureus*, *Staphylococcus epidermidis*, and *Enterococcus faecalis*, while uptake in *Streptococcus agalactiae*, *Streptococcus pyogenes*, and *Listeria monocytogenes* was low. [^18^F]­FMT exhibited similar but lower uptake across Gram-positive bacteria versus [^18^F]­FNT. In contrast, the β-linked glucopyranosyl sorbitol analogs [^18^F]­FLT and [^18^F]­FCT showed negligible uptake in Gram-positive bacteria except for *E. faecalis*, which also accumulated the parent [^18^F]­FDS (Figure 1C).

In Gram-negative bacteria, both α-linked and β-linked glucopyranosyl sorbitol exhibited substantial uptake in *Enterobacter cloacae*, comparable with that of [^18^F]­FDS. Interestingly, the α-linked glucopyranosyl sorbitol analogs [^18^F]­FNT and [^18^F]­FMT showed considerable uptake in *K. pneumoniae*, while β-linked [^18^F]­FLT and [^18^F]­FCT were poorly taken up. No uptake was observed in *Escherichia coli* and *Acinetobacter baumannii* for both α-linked and β-linked glucopyranosyl sorbitol derivatives (Figure 1D). Taken together, these results demonstrate that tracer uptake depends on both the regiochemistry and stereochemistry of [^18^F]­FDS glycosylation, with α-linked derivatives showing more bacterial accumulation than β-linked derivatives.

Given the specific uptake of [^18^F]­FNT in *S. aureus*, we further tested whether uptake was observed in heat-killed bacteria or when competitively blocked with excess (1 mM) nonradioactive [^19^F]­FNT (Figure 1E). The uptake of [^18^F]­FNT was significantly reduced in the presence of nonradioactive [^19^F]­FNT (*P* < 0.0001), while no uptake in heat-killed *S. aureus* (*P* < 0.0001), indicating that [^18^F]­FNT uptake is specific and requires living bacteria. We also tested the uptake of [^18^F]­FNT in clinical isolates of methicillin-sensitive *S. aureus* (MSSA) and methicillin-resistant *S. aureus* (MRSA). [^18^F]­FNT exhibited higher uptake in clinical MSSA and MRSA isolates compared to the reference MSSA strain (Figure 1F, *S. aureus* ATCC 29213, *P* < 0.0001). These findings highlight the potential of α-linked glucopyranosyl sorbitol analogs [^18^F]­FNT and [^18^F]­FMT for detecting infections in vivo caused by *S. aureus*, *S. epidermidis*, and *K. pneumoniae*, which are clinically important pathogens causing bacteremia, endocarditis, and pneumonia.^23^

### [^18^F]­FNT and [^18^F]­FMT Demonstrate Low Background in Uninfected Mice

We selected [^18^F]­FNT and [^18^F]­FMT for further in vivo evaluation in animal models. Prior to in vivo studies, we assessed the in vitro stability of [^18^F]­FNT and [^18^F]­FMT in both mouse and human serum and found that both [^18^F]­FNT and [^18^F]­FMT were intact for up to 120 min at 37 °C. The chemical identities of [^18^F]­FNT and [^18^F]­FMT were confirmed by coinjection with the corresponding nonradioactive ^19^F standards using analytical HPLC (Supporting Information Figures S2 and S3).

To assess the effect of [^18^F]­FDS glycosylation on tissue uptake in vivo, we performed dynamic PET/CT scans for 90 min after the intravenous injection of [^18^F]­FNT, [^18^F]­FMT, and [^18^F]­FDS in uninfected mice, followed by ex vivo analysis. Analysis of dynamic PET/CT images showed that all three tracers exhibited an initial high signal in the body including the heart and lung, followed by urinary excretion (Figure 2A). At 15–30 min postinjection, [^18^F]­FNT and [^18^F]­FMT exhibited lower background signals in nontarget tissues, particularly in the heart, liver, intestine, and kidneys, versus the parent [^18^F]­FDS tracer. Although the ex vivo radioactivity was low for all three tracers in all organs studied, [^18^F]­FNT and [^18^F]­FMT exhibited significantly lower residual activity in organs such as heart, lung, stomach, liver, intestine, kidney, and muscle than did [^18^F]­FDS (Figures 2B and S4), highlighting their favorable clearance and reduced off-target retention compared to [^18^F]­FDS.

**Figure 2:**
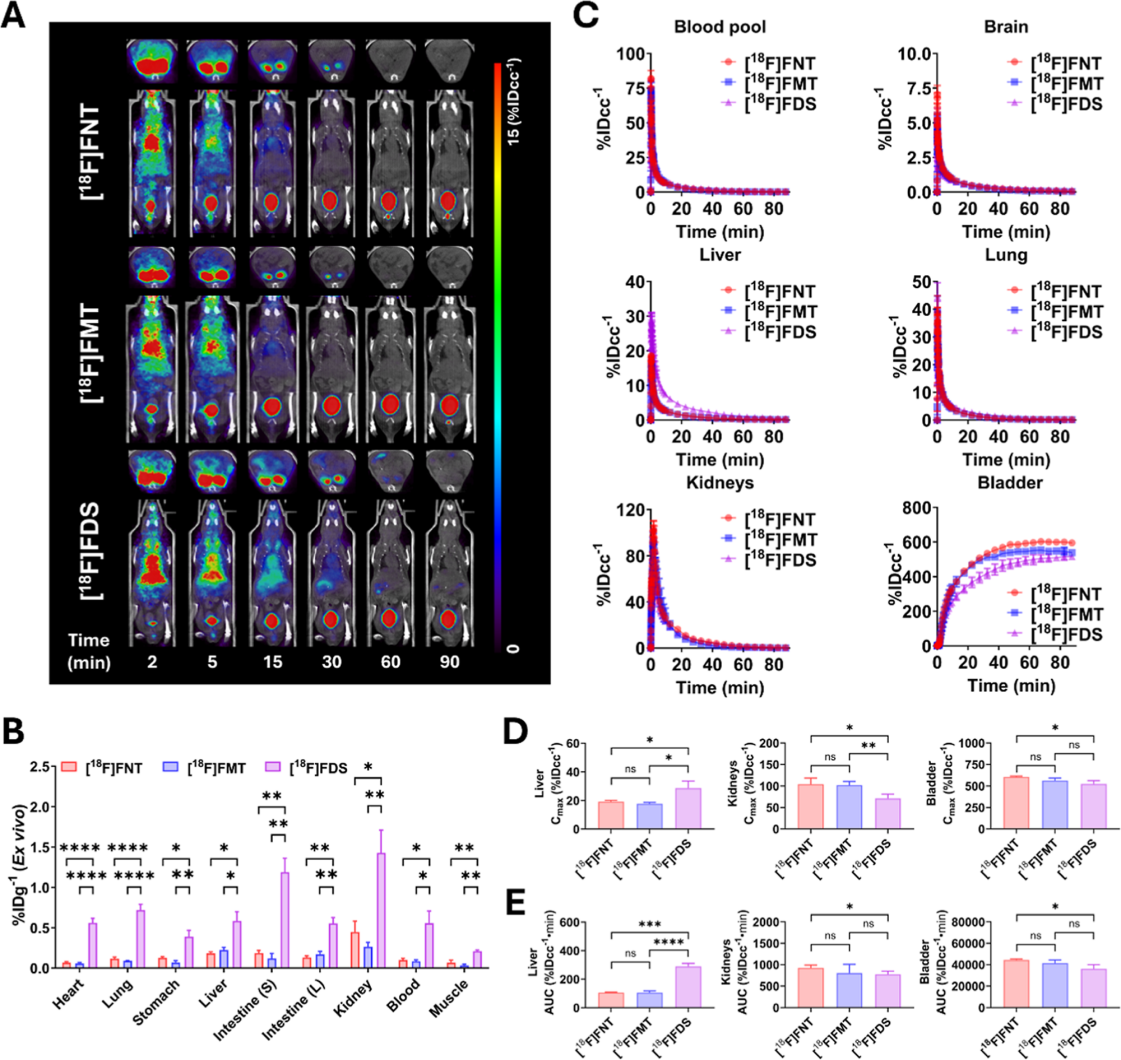
Comparison of whole-body distribution/elimination profiles of [^18^F]­FNT, [^18^F]­FMT, and [^18^F]­FDS in uninfected mice. (A) Representative time–course μPET/CT images of ^18^F tracers in uninfected mice (*n* = 4 for each). (B) Comparison of ex vivo biodistribution of [^18^F]­FNT, [^18^F]­FMT, and [^18^F]­FDS after PET/CT scans. (C) Time–activity curves (TACs) of the blood pool and key organs including brain, lungs, liver, kidneys, and bladder. The data were expressed as the mean ± SEM. (D,E) Comparison of maximum uptake (*C*_max_) and area under the curve (AUC_0→90min_) in liver, kidneys, and bladder. ns = not significant, *P < 0.05, **P < 0.01, ***P < 0.001, and ****P < 0.0001 by one-way ANOVA with Dunnett’s multiple comparisons test (B) and unpaired Student’s *t*-test (D,E).

TACs show the distribution and elimination profiles of all tracers in uninfected mice (Figure 2C). All tracers showed peak uptake in the distribution phase, followed by rapid washout in the elimination phase. [^18^F]­FNT and [^18^F]­FMT showed comparable peak uptake (*C*_max_, % IDcc^–1^) in the liver (19.2 ± 0.9 vs 17.7 ± 0.9; *P* = 0.7079), kidneys (104.5 ± 14.2 vs 102.3 ± 8.4; *P* = 0.9426), and bladder (606.8 ± 9.0 vs 562.7 ± 31.3; *P* = 0.0976). [^18^F]­FDS showed significantly higher peak uptake in liver (28.6 ± 5.0; *P* = 0.0030) and lower peak uptake in kidneys (71.3 ± 9.7; *P* = 0.0039) and bladder (525.5 ± 37.0; *P* = 0.0054) than [^18^F]­FNT. The area under the curve (AUC, % IDcc^–1^·min) of [^18^F]­FNT was not significantly different from that of [^18^F]­FMT but significantly lower than that of [^18^F]­FDS in liver (105.7 ± 4.0 vs 289.4 ± 21.3; *P* < 0.0001) and higher in bladder (44418.3 ± 950.9 vs 36165.8 ± 3961.5; *P* = 0.0083) (Figure 2D,E). The plasma distribution half-life (*T*_1/2α_) of [^18^F]­FNT, [^18^F]­FMT, and [^18^F]­FDS was comparable (0.52, 0.55, and 0.43 min, respectively), whereas the elimination half-life (*T*_1/2β_) of [^18^F]­FNT and [^18^F]­FMT (14.07 and 12.70 min) was shorter than that of [^18^F]­FDS (18.74 min), also supporting the observed rapid clearance and low off-target retention in vivo and ex vivo achieved by glycosidic modification of the sorbitol scaffold.

### In Vivo Studies in Infected Mice Show that the Glycosylated Derivatives [^18^F]­FMT and [^18^F]­FNT Show Low Background Uptake and Specific Uptake in Infected Bacterial Tissues in Two Different Mouse Models of Infection

Given promising low background signals in uninfected mice, we further evaluated [^18^F]­FNT and [^18^F]­FMT in two different clinically relevant mouse models of lung and muscle infections. Based on the dynamic profiles of [^18^F]­FNT and [^18^F]­FMT in uninfected mice (Figure 2), static scans were performed for 20 min between 70 and 90 min postinjection to allow sufficient clearance of tracer from major organs and the blood pool, thereby maximizing target to background signals in infected tissues. As in vitro cultures of *K. pneumoniae* showed robust uptake of both [^18^F]­FNT and [^18^F]­FMT (Figure 1D), we tested their specificity in a well-established *K. pneumoniae* pneumonia murine model.^24^ PET scanning was performed at 24 h postintranasal bacterial installation (Figure 3A). [^18^F]­FDS showed background signals in both uninfected and infected mice within the 0.1–1.0% IDcc^–1^ scale range at 80 min postinjection (mid time). In contrast, [^18^F]­FMT and [^18^F]­FNT showed lower background signals in infected mice than did [^18^F]­FDS, improving image contrast (Figure 3B). The ROI-derived infected/uninfected lung ratios of [^18^F]­FDS, [^18^F]­FMT, and [^18^F]­FNT were approximately 3.2, 6.1, and 5.9-fold, respectively (Figure 3C), also supported by the ex vivo infected/uninfected lung ratio of [^18^F]­FDS (∼1.6-fold), [^18^F]­FMT (∼5.7-fold), and [^18^F]­FNT (∼5.7-fold) (Figure 3D). Post-PET scans, infected lungs were harvested to determine colony forming units (CFUs). No significant differences in bacterial counts were observed among [^18^F]­FDS, [^18^F]­FMT, and [^18^F]­FNT (Figure 3E). To confirm radiotracer specificity, we also evaluated mice inoculated with 10x heat-killed *K. pneumoniae* into lungs (Supporting Information Figure S5A–C). No visible signal was observed in heat-killed *K. pneumoniae* inoculated lungs for both [^18^F]­FNT and [^18^F]­FMT (Supporting Information Figure S5A). The detected activity was comparable to that in uninfected controls (Supporting Information Figure S5B,C). These data indicate that [^18^F]­FNT and [^18^F]­FMT obtained via glycosidic modification of the sorbitol scaffold could provide high-contrast PET images of pulmonary infections.

**Figure 3:**
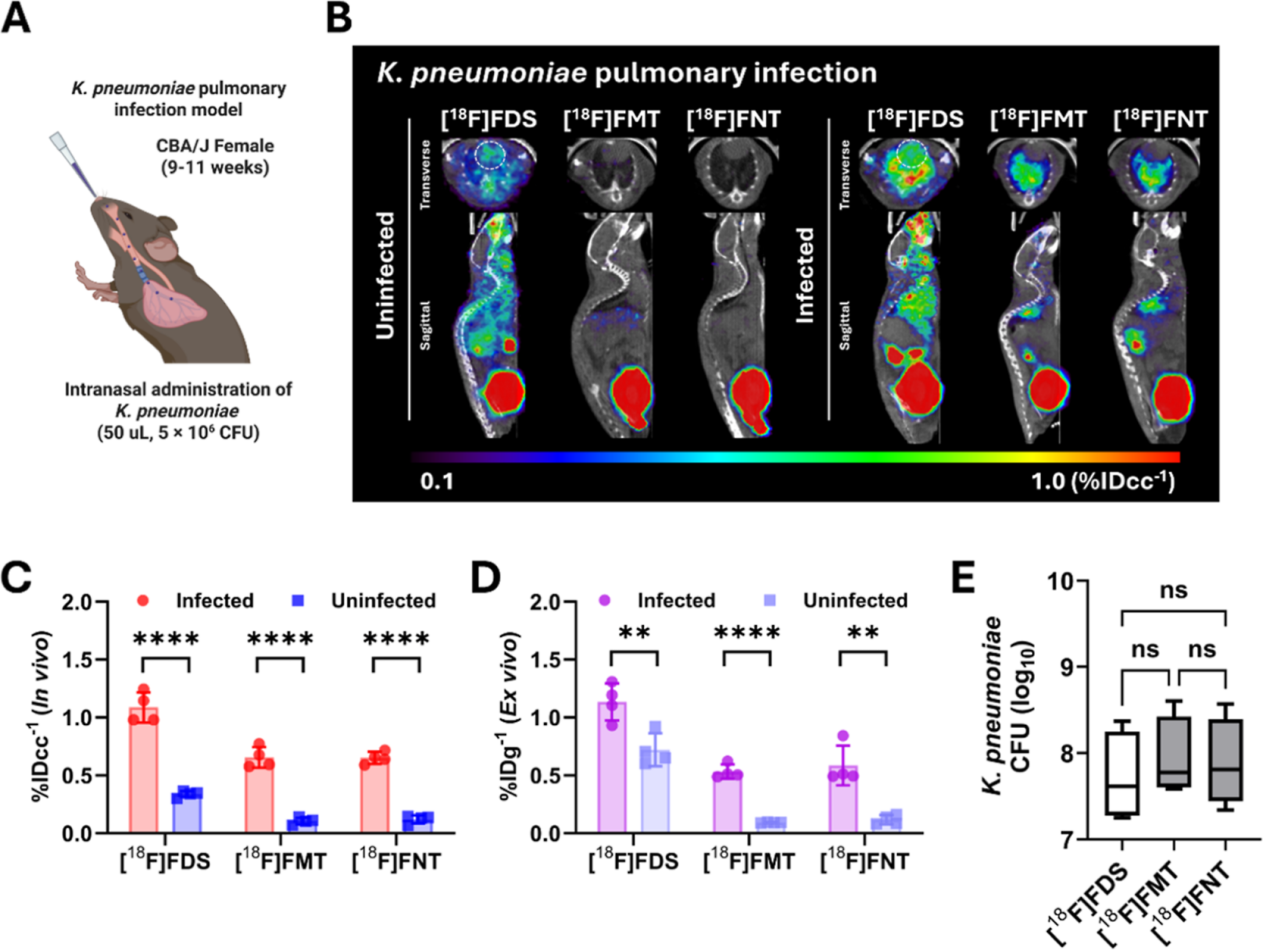
μPET/CT imaging of ^18^F-tracers in a *K. pneumoniae* pulmonary infection model. (A) Schematic illustration (created in BioRender) of the *K. pneumoniae* pulmonary infection murine model. (B) Representative μPET/CT images of [^18^F]­FDS, [^18^F]­FMT, and [^18^F]­FNT in uninfected (left panel) and *K. pneumoniae*-infected (right panel) mice (*n* = 4 for each). The white dashed circles indicate the heart. (C,D) Quantitative analysis of ^18^F-tracers in infected and uninfected lungs, derived from in vivo (C) and ex vivo (D) data, respectively. (E) Bacterial counts in lung homogenates of mice intranasally infected with *K. pneumoniae*. **P < 0.01, ****P < 0.0001 by unpaired Student’s *t*-test. ns = not significant by nonparametric one-way ANOVA.

We next compared [^18^F]­FNT and [^18^F]­FMT PET in a *S. aureus* myositis model. Consistent with our in vitro findings in the reference *S. aureus* strain (ATCC 29213), [^18^F]­FNT showed approximately 4-fold higher uptake in clinically isolated MRSA compared with [^18^F]­FMT (*P* < 0.0001) in vitro (Figure 4A). To compare the in vivo detection sensitivity of [^18^F]­FNT and [^18^F]­FMT in a MRSA myositis model, we injected the left deltoid with live MRSA and the right deltoid with 10x heat-killed MRSA, respectively (Figure 4B). The representative PET/CT imaging at 80 min postinjection (mid time) of [^18^F]­FNT (Figure 4C) showed a clearly visible signal at sites inoculated with live MRSA compared to no detectable signal at sites inoculated with heat-killed MRSA (*P* = 0.0022 for live vs heat-killed MRSA). [^18^F]­FMT also showed a specific signal in live MRSA inoculated sites compared with heat-killed inoculated sites (*P* = 0.0079 for live vs heat-killed MRSA). However, [^18^F]­FMT exhibited a much lower signal than [^18^F]­FNT in live MRSA inoculated sites in both in vivo and ex vivo analyses, consistent with our in vitro findings (Figure 4D,E). The ratio of live/heat-killed *S. aureus* inoculated sites for [^18^F]­FSK was significantly higher than that of [^18^F]­FMT in both in vivo (28-fold vs 6-fold; *P* = 0.0009) and ex vivo (17-fold vs 2.7-fold; *P* = 0.0196) analyses (Figure 4F), indicating high detection sensitivity of [^18^F]­FNT for *S. aureus* infection with an excellent signal-to-background ratio.

**Figure 4:**
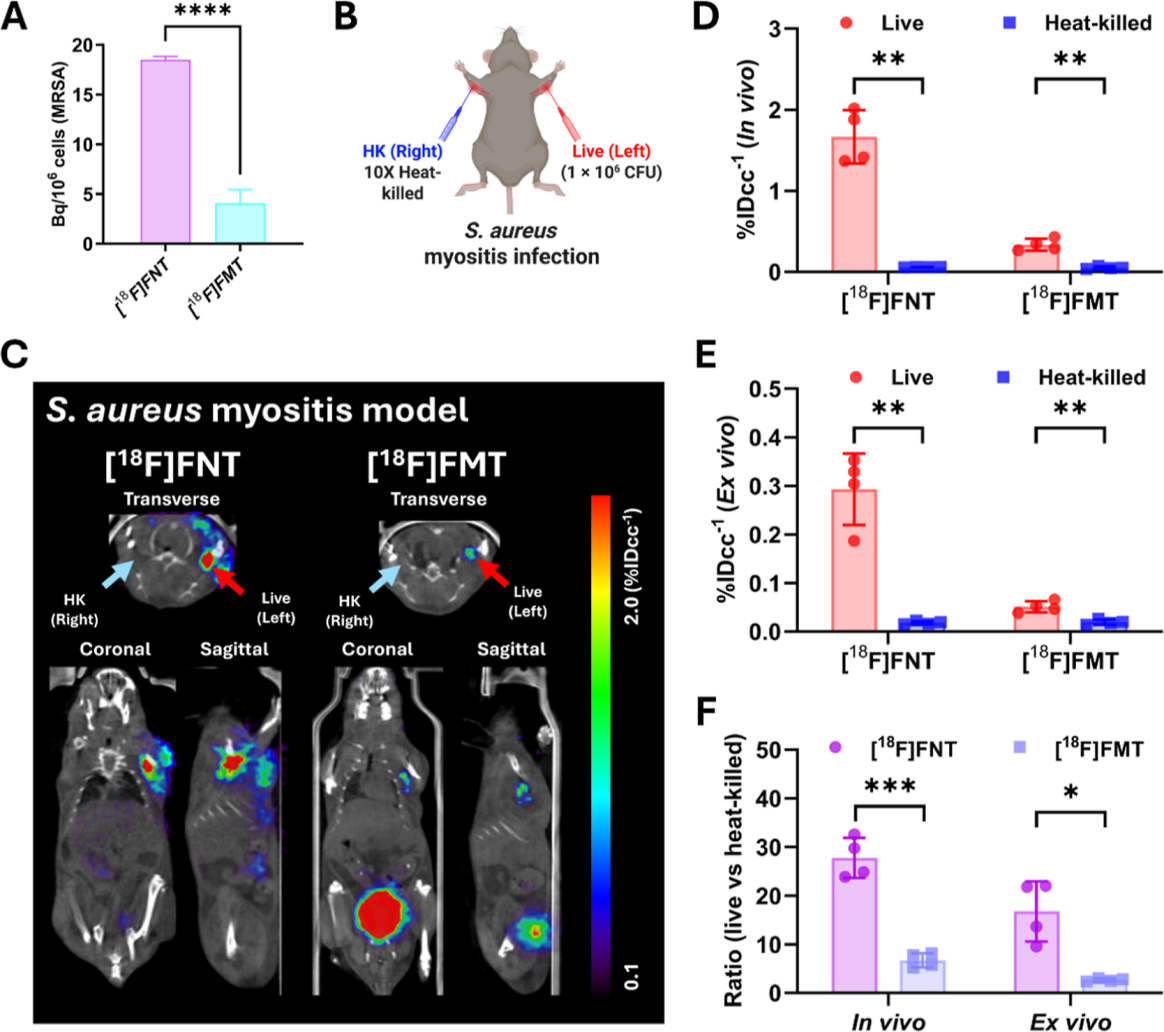
μPET/CT imaging of [^18^F]­FNT and [^18^F]­FMT in a murine myositis model of clinically isolated MRSA infection. (A) Comparison of in vitro uptake of [^18^F]­FNT and [^18^F]­FMT in clinically isolated MRSA. (B) Schematic illustration (created in BioRender) of *S. aureus* (MRSA) murine myositis infection model generation. (C) Representative [^18^F]­FNT and [^18^F]­FMT μPET/CT images of a murine myositis model inoculated with live MRSA in the left deltoid (live, red arrow) and thermally inactivated MRSA in the right deltoid (heat-killed, light blue arrow), respectively. (D,E) Quantitative analysis of [^18^F]­FNT and [^18^F]­FMT in live and heat-killed MRSA inoculated sites, derived from in vivo (D) and ex vivo (E) analysis, respectively. (F) Ratio of radioactivity of live-to-heat-killed MRSA inoculated sites, derived from in vivo and ex vivo data. *P < 0.05, **P < 0.01, ***P < 0.001, and ****P < 0.0001, ns = not significant by paired and unpaired Student’s *t*-tests.

Additionally, we conducted dynamic PET scans of [^18^F]­FNT in a MRSA myositis model as a complementary analysis (Figure 5A–D). As shown in TAC (Figure 5B), both live and heat-killed inoculated sites showed peak uptake at early time points following tracer administration. The uptake at live MRSA inoculated sites decreased gradually and reached a plateau by 90 min, whereas uptake at heat-killed sites rapidly decreased and was negligible by 70 min postinjection of [^18^F]­FNT. Both the ratio of live vs heat-killed and live vs blood pool activity increased over time and reached the highest values at 90 min postinjection (Figure 5C,D).

**Figure 5:**
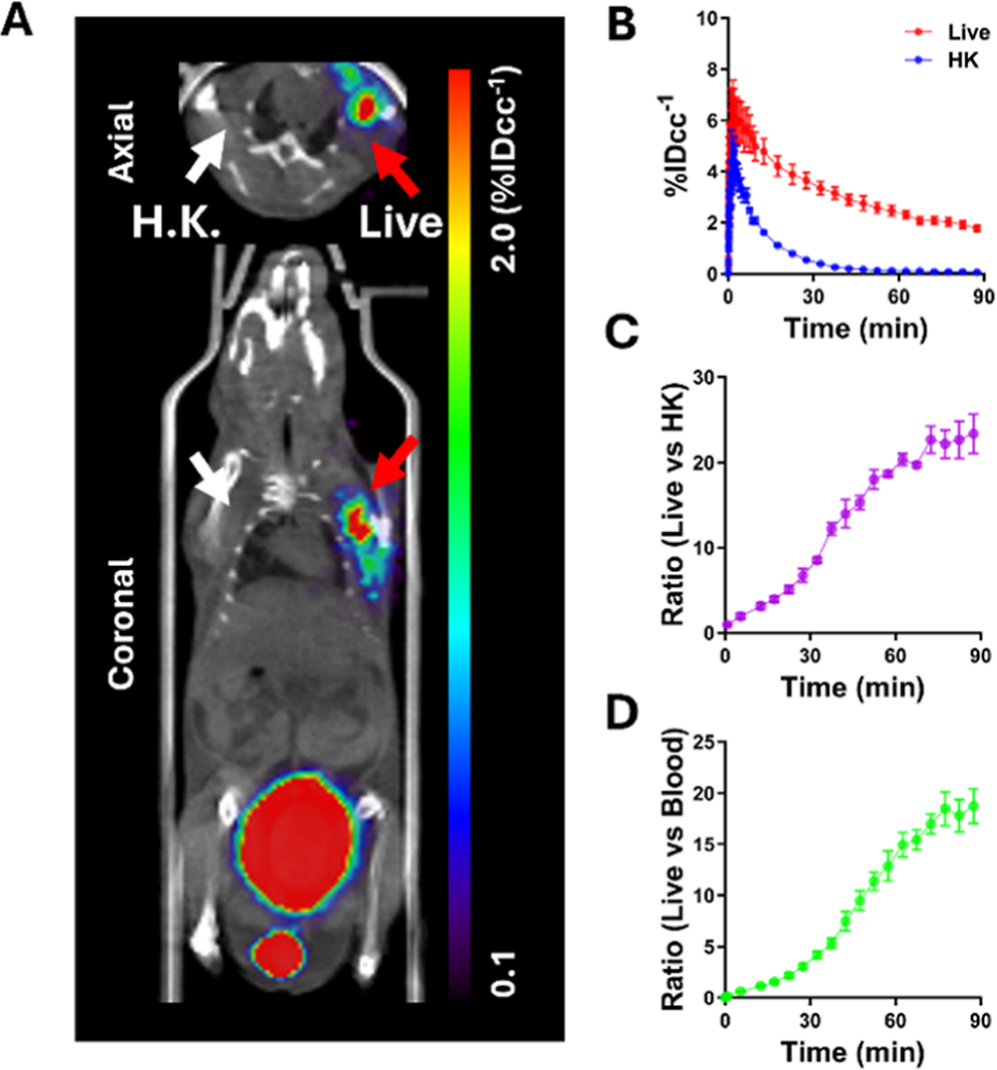
Dynamic μPET/CT scans of [^18^F]­FNT in a murine myositis model of infection using MRSA clinical isolates. (A) Representative [^18^F]­FNT μPET/CT images (summed image between 85 and 90 min p.i.) of a murine myositis model inoculated with live MRSA in the left deltoid (infected, red arrow) and thermally inactivated MRSA in the right deltoid (heat-killed, white arrow). (B) TACs of live MRSA inoculated site (red) and heat-killed MRSA inoculated site (blue). (C,D) ROI-derived ratios of the live/heat-killed (C) and live/blood pool (D).

To further evaluate the detection sensitivity of [^18^F]­FNT, mice were inoculated in the left deltoid with different CFUs of MRSA (10^4^, 10^5^, and 10^6^ CFU) and imaged via PET. Representative PET images of [^18^F]­FNT in variable CFU inoculated mice showed dose-dependent signals at the live MRSA inoculated deltoid from 10^4^ to 10^6^ CFU, with negligible signals at 10^4^ CFU (Figure 6A). Regions of interest (ROI) quantification analysis indicated a CFU-dependent increase of [^18^F]­FNT uptake (Figure 6B), which correlated with the actual bacterial burden determined ex vivo post-imaging study (Figure 6C). Uptake of [^18^F]­FNT at the 10^4^ CFU-inoculated site was not statistically significant from the left deltoid of uninfected controls (*P* = 0.2187).

**Figure 6:**
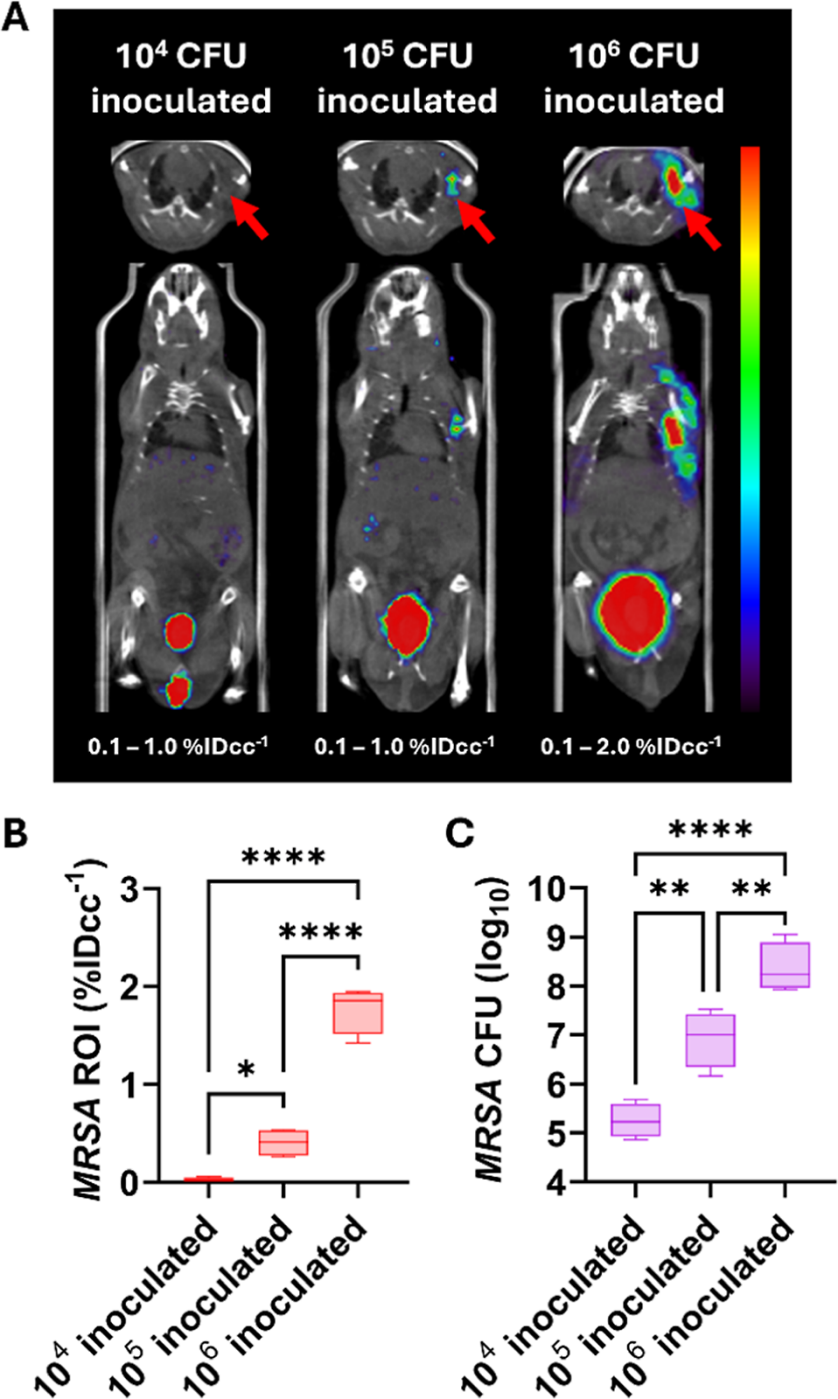
Detection sensitivity analysis of [^18^F]­FNT in a myositis model of MRSA infection. (A) Representative [^18^F]­FNT μPET/CT images with variable CFUs of inoculated MRSA (red arrow) mice. (B) Quantitative in vivo ROI analysis of [^18^F]­FNT uptake in live MRSA inoculated sites. (C) Bacterial counts in live MRSA inoculated tissue homogenates post-imaging study. *P < 0.05, **P < 0.01, and ****P < 0.0001 by nonparametric one-way ANOVA.

### Extrapolated Radiation Dosimetry from Mice Indicates Favorable Human Effective Dose Estimates of [^18^F]­FNT

[^18^F]­FNT demonstrated favorable clearance from the blood pool and uninfected tissues, as well as high uptake in *K. pneumoniae* and *S. aureus* in vitro and in vivo. Based on the comparative results in Figures 3 and 4, [^18^F]­FNT was selected as the lead tracer candidate and further evaluated with dosimetry analysis.

Human dosimetry analysis of [^18^F]­FNT was performed by extrapolating murine biodistribution data using both ICRP60 (Supporting Information Figure S6) and ICRP103 (Figure 7A) models. Among all organs, the urinary bladder was identified as the dose-critical organ (0.1783 ± 0.0314 and 0.1518 ± 0.0262 mSv/MBq for ICRP 60 and 103 model, respectively), followed by the kidneys (0.0753 ± 0.0307 and 0.0751 ± 0.0307 mSv/MBq for ICRP 60 and 103 model, respectively). Overall, the estimated organ-absorbed doses were low (>0.02 mSv/MBq). The calculated human effective doses of [^18^F]­FNT were 0.0163 ± 0.0012 (ICRP 60) and 0.0126 ± 0.0005 (ICRP 103) mSv/MBq, respectively. These values are comparable to the known effective dose of [^18^F]­FDG in the ICRP 103 model (0.017 mSv/MBq). The low effective dose of [^18^F]­FNT, its preclinical performance in infection models, and its high metabolic stability in vivo as indicated by urine samples analysis (Figure 7B–D) support its clinical translation.

**Figure 7:**
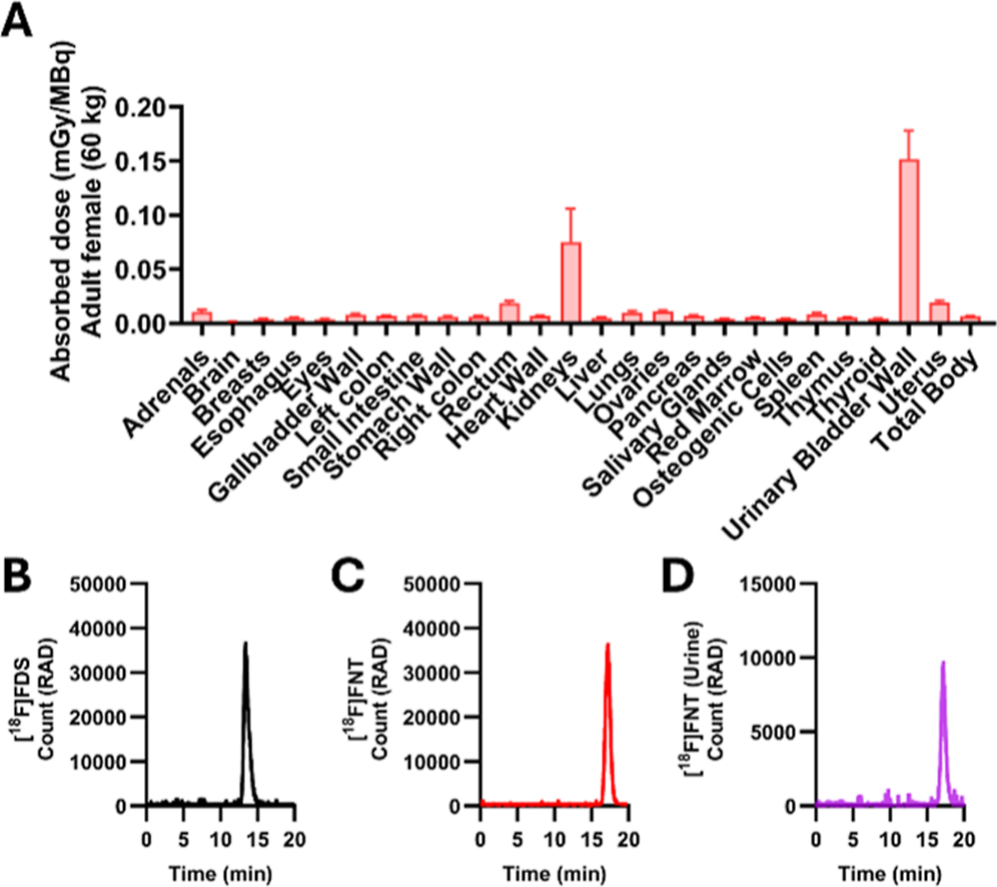
Dosimetry extrapolated to humans and metabolic stability of [^18^F]­FNT. (A) Estimated absorbed dose (mGy/MBq) extrapolated from murine biodistribution data using ICRP103 (adult female, 60 kg). (B–D) Representative radio-HPLC profiles of [^18^F]­FDS (B), [^18^F]­FNT (C), and urine samples collected from healthy mice 90 min following [^18^F]­FNT administration via i.v. (D). [^18^F]­FNT remained intact, with no degradation observed.

## Discussion

In this study, we developed and evaluated α/β-linked glucopyranosyl sorbitol derivatives as novel infection-PET tracers with selective uptake in vitro and in clinically relevant mouse infection models, with favorable clearance and low nonspecific signals in uninfected mice. Based on the panel of bacterial pathogens studied, the specificity of glucopyranosyl sorbitol analogs across pathogens was highly influenced by linkage position (1 → 4 and 1 → 3) and stereochemistry (α and β). Our study highlights that glycosidic configuration plays an important role in bacterial transport and likely reflects underlying differences in the substrate specificity of carbohydrate transporters.

Building upon our previous study on the chemoenzymatic radiosyntheses of ^18^F-disaccharides via reverse phosphorolysis, we applied a simple sodium borohydride-mediated reduction to obtain the corresponding ^18^F-glucopyranosyl-d-sorbitol analogs ([^18^F]­FMT, [^18^F]­FNT, [^18^F]­FCT, and [^18^F]­FLT). In vitro uptake assays revealed distinct selectivity of these analogs across the bacterial pathogens depending on their stereochemistry at the C1 position of the glucanopyranoside. [^18^F]­FCT and [^18^F]­FLT, which are β-linked analogs, showed negligible or lower uptake than that of α-linked analogs, except *E. cloacae*.

Notably, both [^18^F]­FNT and [^18^F]­FMT, which are α-linked glucopyranosyl sorbitol analogs, exhibited significant uptake in *K. pneumoniae*, while β-linked analogs ([^18^F]­FCT and [^18^F]­FLT) showed negligible uptake. No appreciable uptake was observed for both α- and β-linked analogs in *E. coli* and *A. baumannii*. Glycosylation of sorbitol resulted in loss of detection sensitivity in *E. coli*, but [^18^F]­FNT showed significant uptake in Gram-positive bacteria compared to [^18^F]­FDS and other ^18^F-glucopyranosyl sorbitol analogs. While β-linked analogs show poor bacterial sensitivity in the Gram-positive and Gram-negative bacteria tested, these tracers might be evaluated in other microorganisms such as fungi, given that bacterial uptake is closely associated with α-glucan, whereas fungal metabolism and its cell wall are more closely associated with β-glucan.^12^

Although the uptake mechanism of the glucopyranosyl sorbitol analogs remains unclear, our data indicate that tracer uptake is specific to metabolically active bacteria and highly dependent on both glycosidic linkages and stereochemistry. A previous study showed that maltitol is transported by an α-glucoside-specific EIICB component (encoded via *aglA*) of the phosphoenolpyruvate-dependent phosphotransferase system (PTS) and metabolized through 6-phospho-α-glucosidase (encoded by *aglB*).^21,25^ Based on the known metabolism of maltitol, we propose that the α-linked glucopyranosyl sorbitols [^18^F]­FNT and [^18^F]­FMT may undergo a similar metabolic process via the α-glucoside PTS system and 6-phospho-α-glucosidase, potentially explaining the selective uptake in *K. pneumoniae* versus β-linked tracers. However, further validation including transposon mutant analyses are required to fully understand the uptake mechanism of ^18^F-glucopyranosyl-d-sorbitol analogs.

It is worth noting that [^18^F]­FNT and [^18^F]­FMT exhibited rapid and favorable renal clearance with minimal hepatobiliary accumulation and retention in uninfected mice. Although the β-linked analogs [^18^F]­FLT and [^18^F]­FCT revealed poor bacterial sensitivity across the tested pathogens, we also characterized their in vivo behavior using dynamic PET, followed by ex vivo biodistribution (Supporting Information Figures S7A and S8). Similar to the α-linked analogs, both β-linked analogs also showed peak uptake in major organs (<1 min), followed by rapid and dominant excretion through the bladder (Supporting Information Figure S7B). The pharmacokinetic parameters (*C*_max_ and AUC) of β-linked analogs were not significantly different from those of [^18^F]­FNT, indicating that structural differences in glycosidic linkages do not significantly influence in vivo behavior in uninfected mice (Supporting Information Figure S9).

A potential drawback of sugar or sugar alcohol tracers is their susceptibility to nonspecific accumulation in the liver and gastrointestinal tract due to endogenous metabolic activity.^26^ This concern is especially relevant for [^18^F]­FDG-based tracers, as their metabolic degradation may regenerate [^18^F]­FDG, which could potentially be reabsorbed and further accumulate in host tissues, including the myocardium.^27^ This degradation was not observed for any [^18^F]­FDS-derived glucopyranosyl-Dsorbitol analog studied. Our findings suggest that glycosidic modification of the fluorine-18-labeled sugar alcohol [^18^F]­FDS represents a useful strategy to reduce off-target retention and maximize imaging contrast.

Direct comparison of in vivo studies is limited by technical differences such as imaging protocols and bacterial strains; the target-to-nontarget ratio of [^18^F]­FNT in *S. aureus* infected mice (∼28-fold) was high versus that of previously reported *S. aureus* sensitive PET tracers such as D-[^11^C]­Met (∼10-fold),^28^ 2-[^18^F]­F-PABA (∼8-fold),^29^ D-[3-^11^C]­Ala (∼4-fold),^30^ 2-[^18^F]­F-ENB (∼17-fold),^31^ 2-[^18^F]­F-NB (∼17-fold),^31^ [^18^F]­TZ4877 (∼1.3-fold),^32^ D-[5-^11^C]-Gln (∼2.3-fold),^33^ ^18^F-NTRP (∼2.3-folds),^34^ ^18^F-NCPR (∼2.3-fold),^34^ [^18^F]­FMtl (∼4-fold),^35^ [^18^F]­FDM (∼6-fold),^12^ [^18^F]­FSK (∼7-fold),^12^ [^18^F]­FMA (∼7-fold),^36^ [^11^C]­PABA (∼28-fold),^37^ D-[^18^F]­FAla (∼2-fold),^38^ and D-[^18^F]­FAla-d_3_ (∼2.4-fold).^38^ Taken together, our findings indicate the potential of [^18^F]­FNT in imaging bacterial infections with high specificity, favorable clearance, and excellent target-to-background contrast.

In conclusion, we investigated the impact of regioselective and stereoselective modification of the infection-targeted PET radiotracer [^18^F]­FDS to α/β ^18^F-glucopyranosyl-d-sorbitol analogs. These analogs could be efficiently produced from reverse phosphorolysis of clinical [^18^F]­FDG to fluorine-18-labeled disaccharides that were subsequently reduced using NaBH_4_. Our data demonstrate that the glycosidic linkage and configuration of ^18^F-sugar alcohols can markedly influence bacterial specificity, improve tissue clearance, and reduce off-target retention compared to the parent sugar alcohol [^18^F]­FDS. The metabolic stability of [^18^F]­FNT and [^18^F]­FMT offers potential advantages over several other reported tracers, for example, [^18^F]­FDM and [^18^F]­FSK, based on their potential degradation by α-glucosidases activity in humans and carbon-11-labeled tracers, for example, [^11^C]­PABA, which would be difficult to apply in the acute setting due to their short half-lives. These findings also highlight the use of glycosylation to improve PET tracer performance, a strategy more frequently employed in pharmacotherapy. Although β-linked analogs exhibited negligible bacterial uptake, [^18^F]­FCT and [^18^F]­FLT may also hold promise for imaging fungal infections, particularly those involving β-glucan-associated metabolic pathways. These new imaging tools and their rapid radiosynthesis from [^18^F]­FDG will transform the way PET radiotracers are considered and used in the acute care setting.

## Experimental Section

### Reagents and Equipment

All reagents and solvents were purchased from Sigma-Aldrich or TCI and used without further purification. Nuclear magnetic resonance (NMR) spectra were acquired by using a Bruker Advance III HD 400 MHz instrument at the UCSF Nuclear Magnetic Resonance Laboratory. Mass analysis was performed at the University of California, Berkeley Spectrometry Facility. [^18^F]­FDG were produced in the UCSF radiopharmaceutical facility. Radioactivity was measured by using a Hidex Automatic Gamma Counter (Hydex Oy). Radio TLC analysis was carried out by using a radio TLC scanner (Bioscan AR200, Bioscan Inc.). μPET/CT imaging was conducted using a nanoScan μPET/CT (Mediso). Bacterial strains and culture conditions are provided in Table S1. The syntheses of ^19^F-standards for radiopharmaceutical characterization are described in the Supporting Information.

### Radiochemistry

[^18^F]­FDG was produced at the UCSF radiopharmaceutical facility and used for the described steps without further purification. [^18^F]­FDS was prepared according to the literature using NaBH_4_.^22^ [^18^F]­FNT, [^18^F]­FMT, [^18^F]­FLT, and [^18^F]­FCT were synthesized by applying the same reduction protocol described for [^18^F]­FDS, starting from the corresponding disaccharides ([^18^F]­FSK, [^18^F]­FDM, [^18^F]­FDL, and [^18^F]­FDC) which were prepared following our previously established protocol.^12^ Briefly, [^18^F]­FDG in citrate buffer (∼30 mCi in 0.4 mL) was added to a vial containing phosphorylase enzyme (∼5 units) and 6 mg of α-glucose-1-phosphate (for [^18^F]­FDL and [^18^F]­FDC) or β-glucose-1-phosphate (for [^18^F]­FSK and [^18^F]­FDM) and then reacted at 37 °C for 20 min. ^18^F-disaccharides were isolated by semiprep HPLC (YMC-Pack Polyamine II 250 × 10.0 mm I.D. S-5um, 72.5% MeCN/Water, flow rate: 4.0 mLmin^–1^). A Sep-Pak Plus NH_2_ cartridge was then used to remove acetonitrile. Aqueous solutions of ^18^F-disaccharides (0.5 mL) were transferred to vials containing 4 mg of NaBH_4_ and reacted at 40 °C for 20 min. After the pH was adjusted to 7.4, the resulting solutions were passed through *N*-alumina Sep-Pak cartridges, followed by filtration. The complete conversion of ^18^F-disaccharides to ^18^F-glucopyranosyl-d-sorbitols was confirmed by radio-TLC. The identity of [^18^F]­FNT and [^18^F]­FMT was verified by coinjection with the ^19^F standard by analytical HPLC (YMC-Pack Polyamine II 250 × 4.6 mm I.D. S-5um; 77.5% MeCN/Water; 1 mL min^–1^).

### In Vitro Assay

In vitro evaluation of ^18^F-tracers was conducted according to our established procedure.^36^ Briefly, bacteria were incubated overnight at 37 °C, subcultured into a fresh medium at an initial OD_600_ of 0.05, and then allowed to reach the mid log phase (OD_600_ ∼ 0.4). The cultures were subsequently incubated with ^18^F-tracers (final concentration: 0.1 MBq/mL) at 37 °C for 90 min. Subsequently, 300 μL aliquots of these cultures were centrifuged at 13,200 rpm for 6 min. The resulting pellets were washed with 300 μL of phosphate-buffered saline (PBS). The radioactivity of the pellets and supernatants was measured using a gamma counter. Uptake values were normalized to colony-forming units (CFUs).

### Animal Infection Model

All animal procedures were conducted in accordance with UCSF institutional guidelines and were approved by the Institutional Animal Care and Use Committee (IACUC). Female CBA/J mice (9 and 11 weeks old) were used for the study. Pulmonary infection and bacterial myositis models were established based on previously published protocols.^24,36^ For pulmonary infection, 50 μL of *K. pneumoniae* (5 × 10^6^ CFU) or 10× heat-killed *K. pneumoniae* were administered intranasally under anesthesia 24 h prior to imaging using PET. After PET scans, infected lungs were homogenized in PBS and plated on LB agar. Plates were incubated overnight at 37 °C to quantify the bacterial burden. For the bacterial myositis model, mice were intramuscularly injected with either 1 × 10^6^ CFUs of live *S. aureus* (MSSA or MRSA) or 1 × 10^7^ CFUs of heat-killed bacteria (50 μL) into the left and right deltoid muscles, respectively, 10 h prior to imaging using PET. Immediately following all imaging studies, target tissues were harvested, and radioactivity was measured using a gamma counter.

### PET/CT Imaging and Data Analysis

μPET/CT imaging was conducted using a nanoScan μPET/CT instrument (PET123S/CT1512, Mediso). Mice were anesthetized with 2% isoflurane/oxygen for intravenous administration of ^18^F-tracers (∼5 MBq in 150 μL) via tail vein and maintained during data acquisition. PET data of uninfected mice were acquired in the list mode for 90 min, starting immediately after ^18^F-tracer administration, followed by a CT scan. The PET data were reconstructed into three-dimensional volumes and coregistered with CT images using manufacturer-provided software in 53 dynamic frames (15 × 2 s, 6 × 5 s, 6 × 10 s, 4 × 30 s, 6 × 30 s, and 16 × 300 s). PET data of infected mice were acquired for 20 min, starting at 70 min postinjection of ^18^F-tracer (mid time 80 min), followed by a CT scan. The list mode PET data were reconstructed into three-dimensional volumes in a single static frame. All PET scans were corrected for ^18^F decay, dead time, and random coincidences and reconstructed into a 160 × 160 × 184 matrix with voxel size 0.8 mm using a three-dimensional ordered subject expectation maximization (OSEM) algorithm (40 iterations, 1 subset). Both attenuation and scatter corrections were applied to PET reconstructions using the coregistered CT. ROIs were drawn manually around the brain, lungs, liver, kidney, and urinary bladder using Amide software to generate image-derived TACs.^39^ For the estimation of blood pool radioactivity over time, ROIs were drawn on the left atrium. To estimate the plasma half-life of tracers, the TAC of the blood pool was fitted to a two-compartment model using a biexponential decay function: *C*_*t*(*t*)_ = *Ae* ^–α*t*^ + *Be* ^*‑*β*t*^, where *C*_*t*(*t*)_ is the tracer concentration (% IDcc^–1^) at time (*t*), A and B are the intercepts, and α and β are the distribution and elimination rate constants, respectively.^40,41^ Quantitative uptake values are expressed as % IDcc^–1^, representing the percent of the total injected dose per cubic centimeter. For the *S. aureus* myositis model, identical volume ROIs were drawn corresponding to the peak uptake of [^18^F]­FNT at the live *S. aureus* inoculated site and heat-killed *S. aureus* inoculated site, respectively.

### Radiation Dose Estimation

A dosimetry study was carried out in female CBA/J mice (*n* = 4, 9–11 weeks). Human organ and whole-body effective doses were extrapolated from the mice biodistribution data according to our previous report.^23^ For each organ, absorbed and equivalent doses (mGy/MBq or mSv/MBq), as well as whole-body effective doses (mSv/MBq), were calculated from murine data. Dose estimations were performed with OLINDA software (v1.1 and 2.0 with ICRP 60 and 103 tissue weighting factors).^42,43^

### Statistical Analysis

All statistical analyses and graph generation were performed using GraphPad Prism v10 software (GraphPad Software Inc.). One-way ANOVA or paired/unpaired Student’s *t*-tests were used to assess statistical significance. *P* < 0.05 was considered statistically significant. All data were expressed as mean ± standard deviations (s.d.), unless otherwise specified.

## Supplementary Material



## Notes

Synthesis of [^19^F]­FNT and [^19^F]­FMT, radio-TLC profile, serum stability, ex vivo biodistribution data, and bacterial strains and growth conditions used for this study (PDF)
