# Supplementary material for: Regioselective Glycosylation of Fluorine-18-Labeled Sorbitol for Enhanced Bacterial Detection In Vivo Using PET
Source: JACS Au. 2025 Dec 1;5(12):6189–99. doi: 10.1021/jacsau.5c01153 (PMC12728616; doi:10.1021/jacsau.5c01153)
Supplement: Supplementary file 1 [file au5c01153_si_001.pdf]

Supporting Information for

Regioselective glycosylation of fluorine-18 labeled sorbitol for enhanced bacterial detection *in vivo* using PET.

Sang Hee Lee[1], Jung Min Kim[1], Marina López-Álvarez[1], Alexandre M. Sorlin[1], Mohammad Yaqoob Bhat[1], Joseph Blecha[1], Robert R. Flavell[1], Youngho Seo[1,2,3], Joanne Engel[4], Michael Ohliger[1,5], and David M. Wilson[1*]

[1]Department of Radiology and Biomedical Imaging, University of California, San Francisco, San Francisco, CA 94158, USA
[2]Department of Nuclear Engineering, University of California, Berkeley, California
[3]UCSF Helen Diller Family Comprehensive Cancer Center, San Francisco, California
[4]Department of Medicine, University of California, San Francisco, San Francisco, CA 94158, USA
[5]Department of Radiology, Zuckerberg San Francisco General Hospital, San Francisco CA 94110, USA

* David Wilson, M.D., Ph.D.
Email: david.m.wilson@ucsf.edu

This PDF file includes:

Chemistry
^{1}H, ^{13}C, and ^{19}F NMR spectra
Figures S1 to S4
Tables S1

Chemistry

General synthesis of non-radioactive glycopyranosyl-D-fluorosorbitols

An aqueous solution (2 mL) of 2-deoxy-2-fluoro-maltose or 2-deoxy-2-fluoro-sakebiose (30 mg) was added to a vial containing $NaBH_4$ (15 mg) and stirred at room temperature for 1 h. After adjusting the pH to 7.4, the resulting mixture was passed through an N-alumina Sep-Pak cartridge. The resulting solution was lyophilized using freeze-drying to afford the desired glycopyranosyl-D-fluorosorbitols in quantitative yields.

4-O-α-glucopyranosyl-D-2-deoxy-2-fluorosorbitol (fluoromaltitol, FMT)

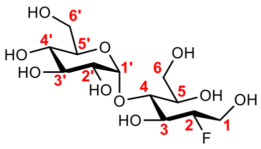

FMT
$\alpha(1 \rightarrow 4)$ **bond**

White powder (quantitative). ^{1}H NMR (400 MHz, D_2O) δ 5.04 (C1’, d, J = 3.9 Hz, 1H), 4.89-4.81 (C2, m, 1H), 4.08-4.00 (C3, m, 1H), 4.00-3.91 (C5, C3’, m, 2H), 3.89 - 3.64 (C1, C4, C5’, C6’ m, 6H), 3.61 (C6, dd, J = 11.7, 7.0 Hz, 2H), 3.55 - 3.47 (C2’, 1H), 3.38 (C4’ t, J = 9.5 Hz, 1H); ^{13}C NMR (100 MHz, D_2O) δ 100.12 (C1’), 93.91 (C2, d, *J* = 172.3 Hz), 80.18 (C4), 72.77 (C3’), 72.40 (C5’), 72.33 (C5), 71.62 (C2’), 69.86 (C3’, d, *J* = 18.2 Hz), 69.37 (C4’), 62.22 (C6), 61.15 (C1’, d, *J* = 22.2 Hz), 60.35; ^{19}F NMR (376 MHz, D_2O) δ -203.92. MS (EI-Neg) m/z $[M+Cl]^-$ calcd for $C_{12}H_{23}FO_{10}Cl^-$ 381.0969, found 381.0966.

3-O-α-glucopyranosyl-D-2-deoxy-2-fluorosorbitol (fluoronigeritol, FNT)

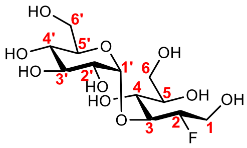

FNT
$\alpha(1 \rightarrow 3)$ **bond**

White powder (quantitative). ^{1}H NMR (400 MHz, D_2O) δ 5.09 (C1’, d, J = 3.9 Hz, 1H), 4.90 – 4.70 (C2, m, 1H), 4.18 (C3, dd, J = 13.0, 7.1 Hz, 1H), 3.95 – 3.73 (C1, C4, C6, C5’ C6’, m, 6H), 3.72 – 3.57 (C5, C6, C3’, m, 4H), 3.51 (C2’, dd, J = 10.0, 3.8 Hz, 1H), 3.39 (C4’, t, J = 9.4 Hz, 1H); ^{13}C NMR (100 MHz, D_2O) δ 100.23 (C1’), 94.69 (C2, d, *J* = 172.6 Hz), 77.06 (C3, d, *J* = 21.1 Hz), 72.74 (C3’), 72.12 (C5’), 71.76 (C2’), 69.91 (C4), 69.68 (C5), 69.33 (C4’), 62.86 (C6), 60.78 (C1, d, *J* = 21.0 Hz), 60.30 (C6’); ^{19}F NMR (376 MHz, D_2O) δ -195.71. MS (EI-Neg) m/z $[M+Cl]^-$ calcd for $C_{12}H_{23}FO_{10}Cl^-$ 381.0969, found 381.0967.

^{1}H, ^{13}C, and ^{19}F NMR spectra

4-O-α-glucopyranosyl-D-2-deoxy-2-fluorosorbitol ^{1}H NMR

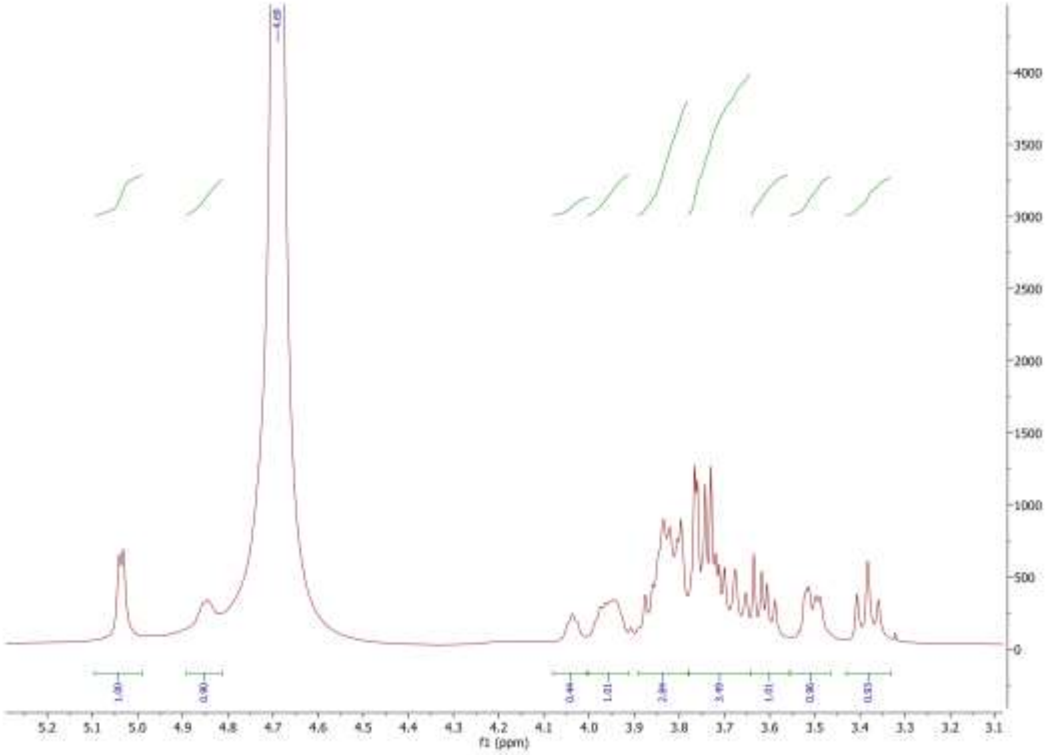

4-O-α-glucopyranosyl-D-2-deoxy-2-fluorosorbitol ^{13}C NMR

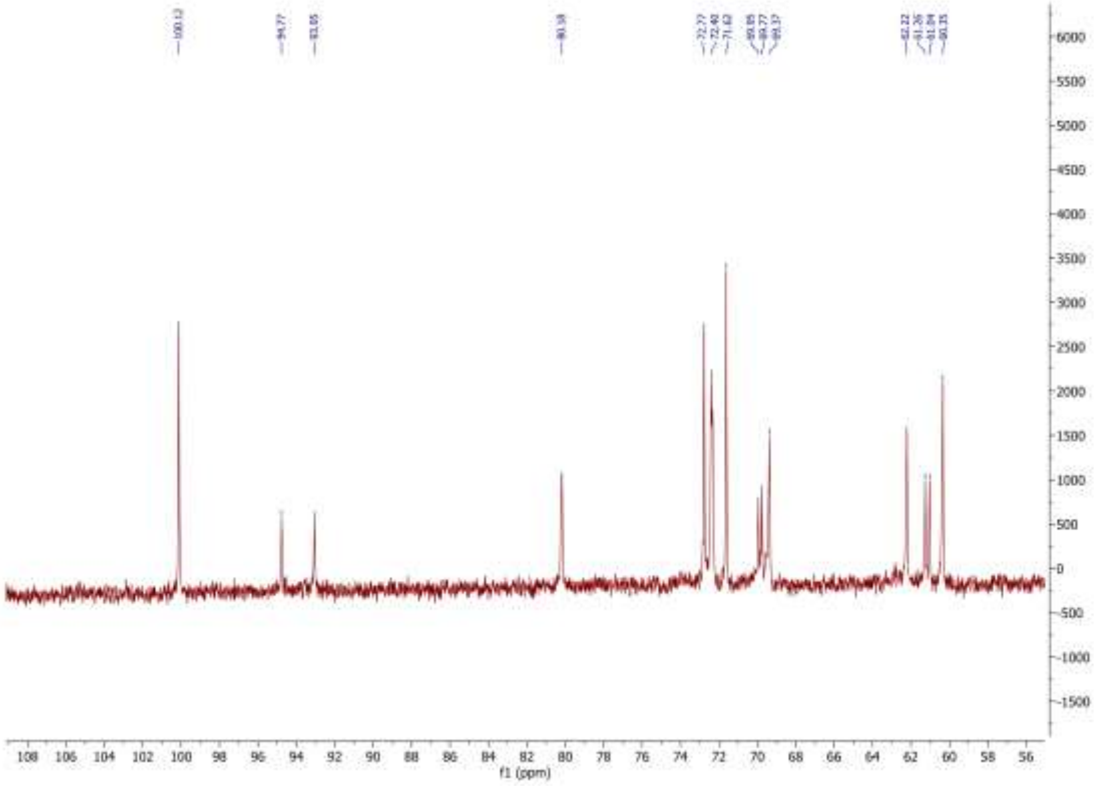

4-O-α-glucopyranosyl-D-2-deoxy-2-fluorosorbitol ^{19}F NMR

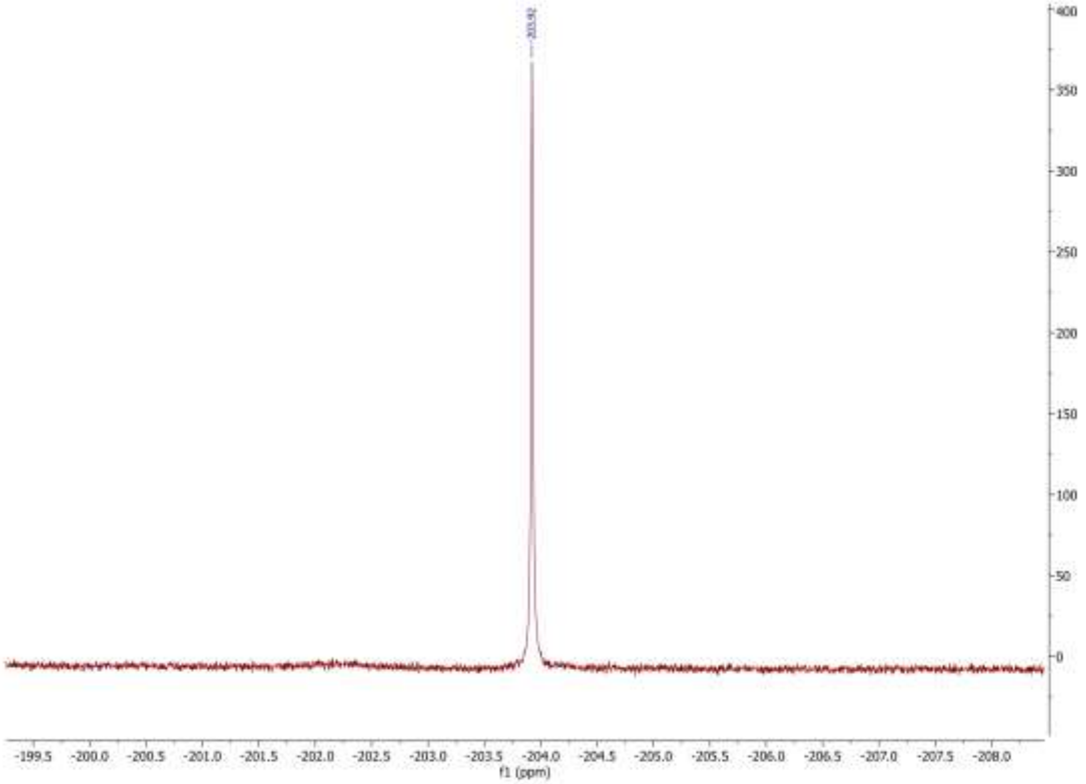

4-O-α-glucopyranosyl-D-2-deoxy-2-fluorosorbitol HSQC NMR

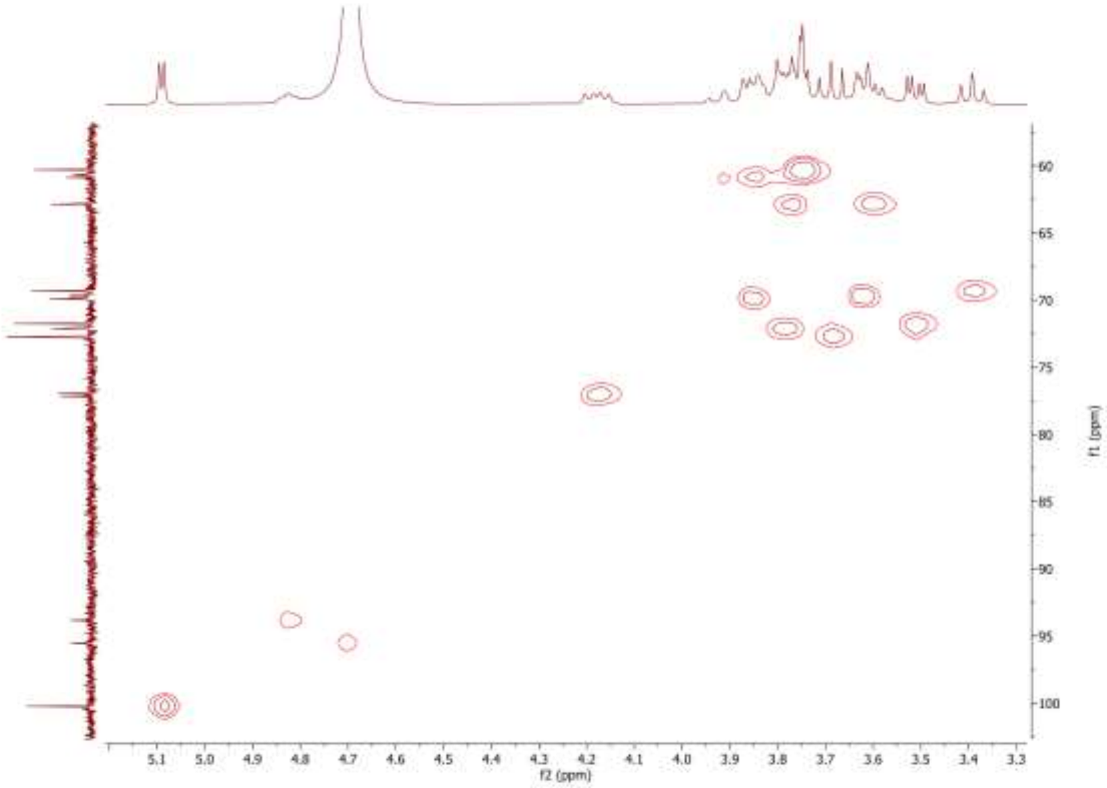

3-O-α-glucopyranosyl-D-2-deoxy-2-fluorosorbitol ^{1}H NMR

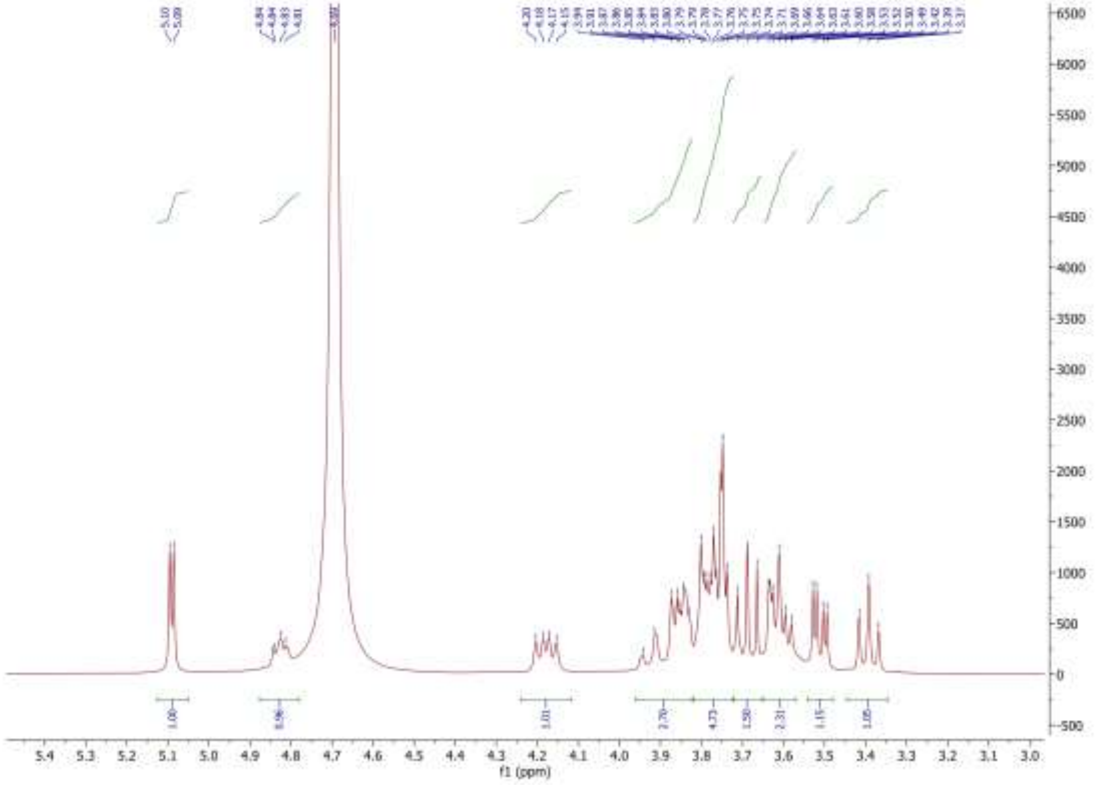

3-O-α-glucopyranosyl-D-2-deoxy-2-fluorosorbitol ^{13}C NMR

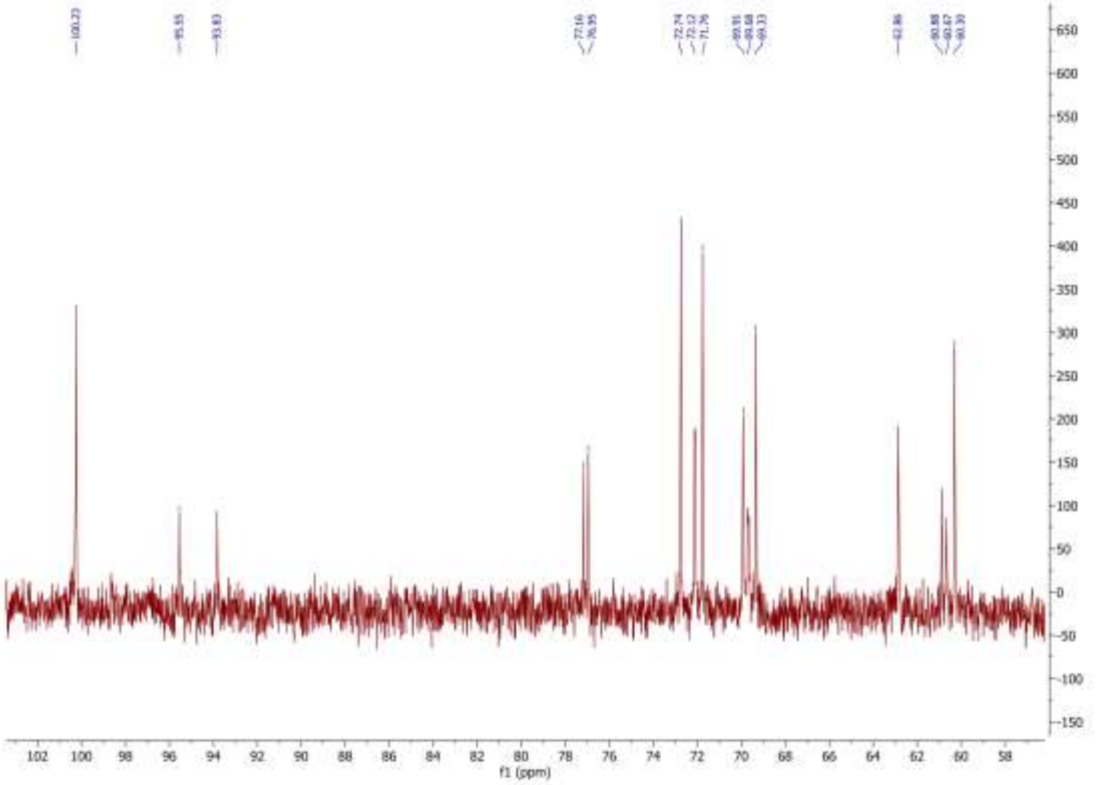

3-O-α-glucopyranosyl-D-2-deoxy-2-fluorosorbitol ^{19}F NMR

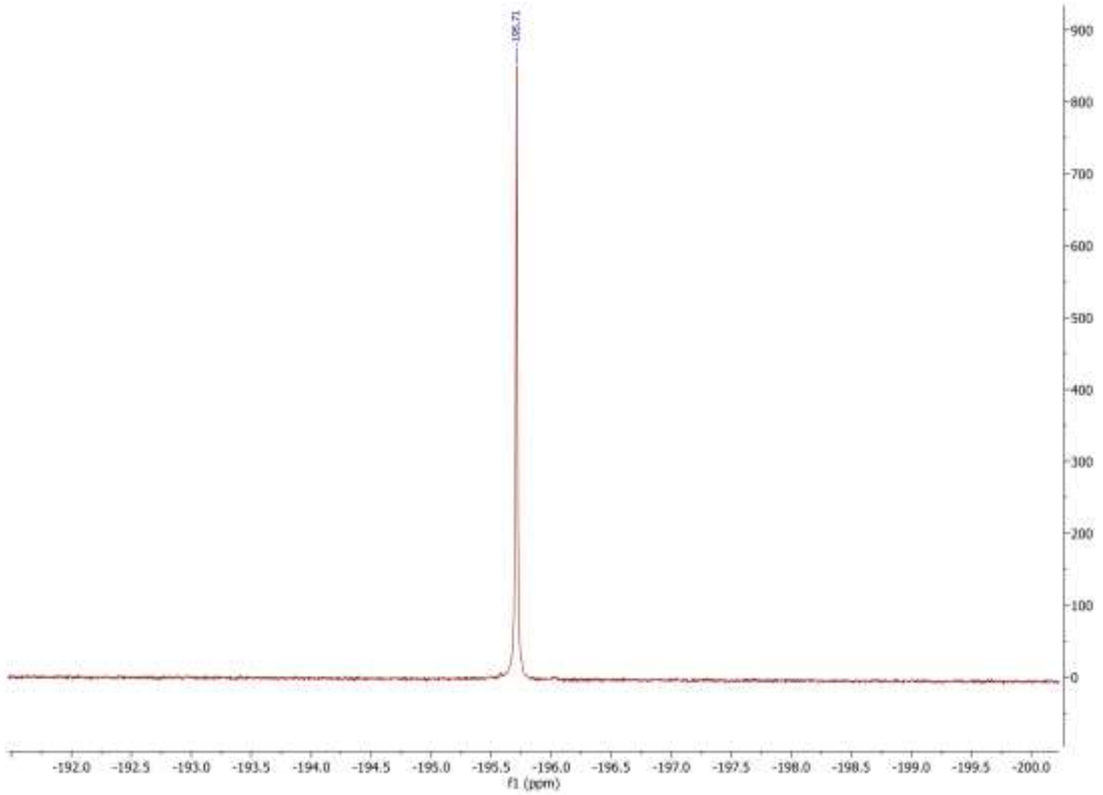

3-O-α-glucopyranosyl-D-2-deoxy-2-fluorosorbitol HSQC NMR

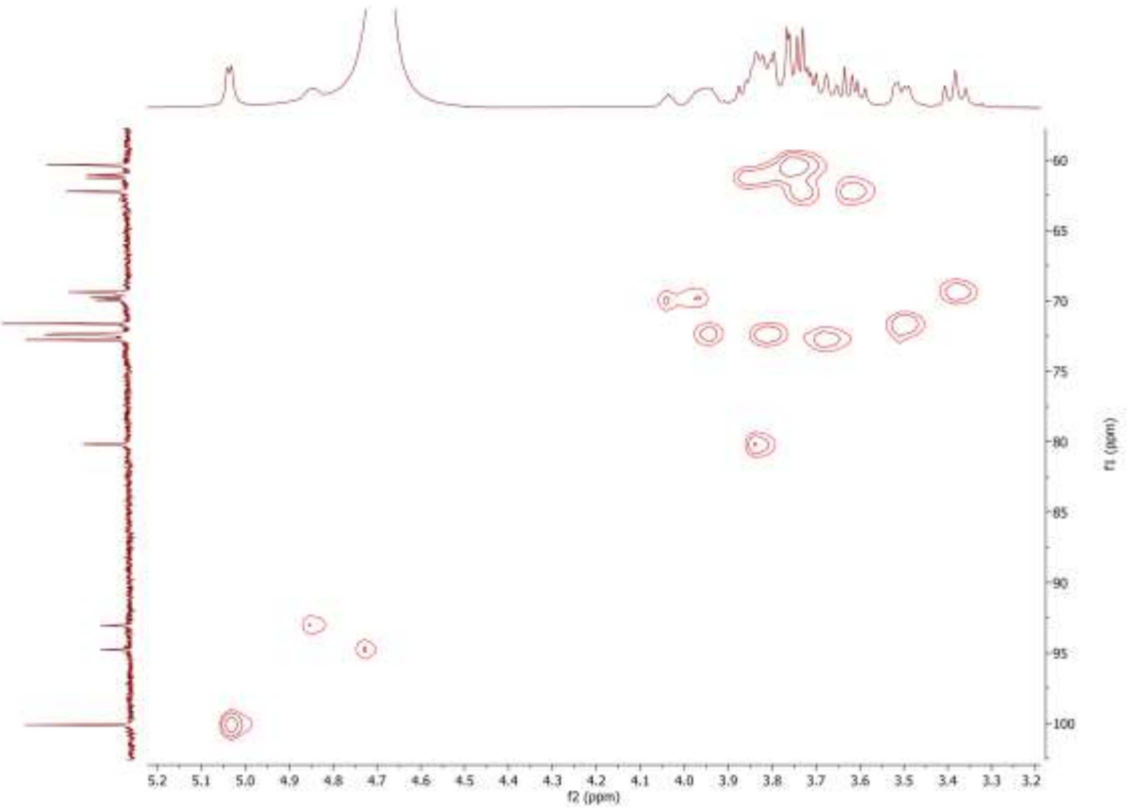

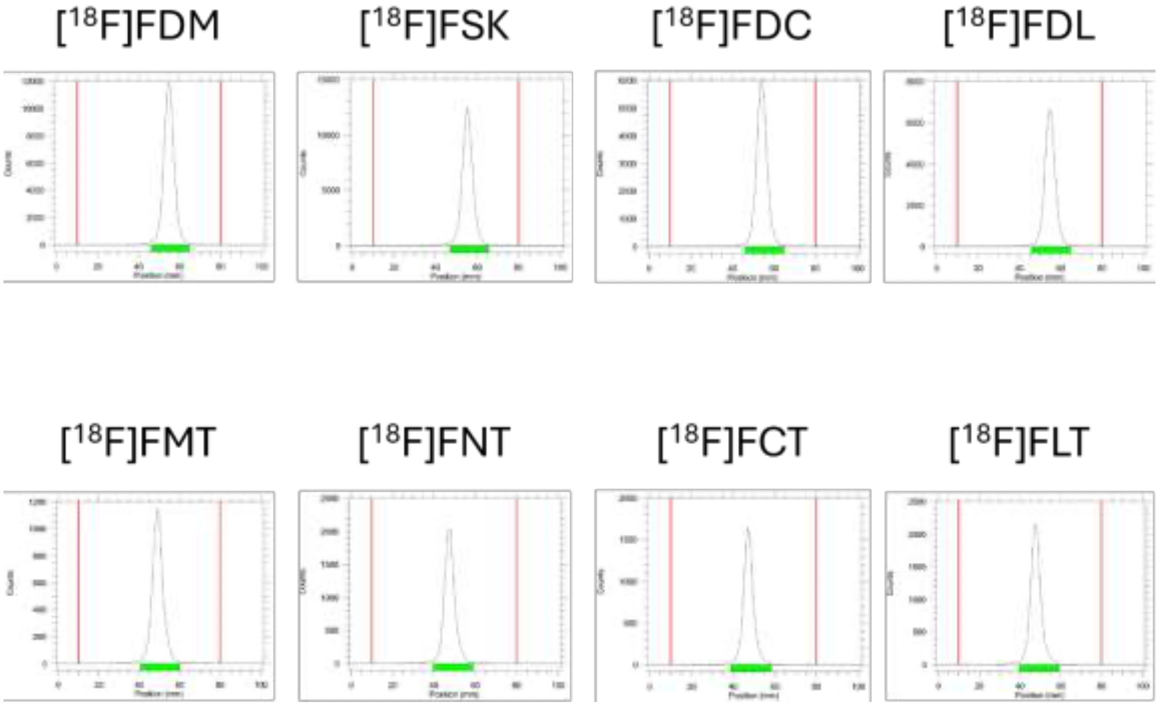

Fig. S1. Radio-TLC profiles of ^{18}F-disacchardes ([^{18}F]FSK, [^{18}F]FDM, [^{18}F]FDL, [^{18}F]FDC; top) and ^{18}F-glucopyranosyl-D-sorbitols ([^{18}F]FNT, [^{18}F]FMT, [^{18}F]FLT, [^{18}F]FCT; bottom). TLCs were developed using 70% acetonitrile/water as a mobile phase.

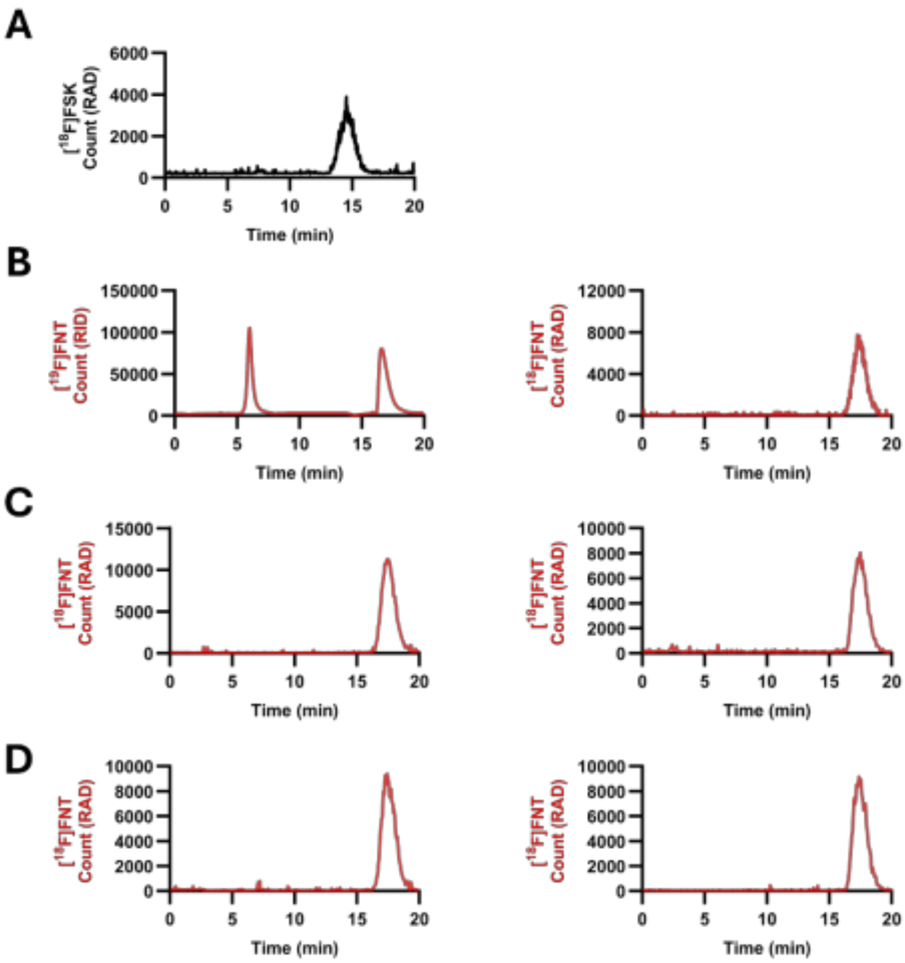

Fig. S2. Analytical-HPLC analysis (YMC-Pack Polyamine II I.D. S-5 μm 250x4.6 mm; 80 % MeCN/Water; 1 $mLmin^{-1}$) of (A) [^{18}F]FSK, (B) [^{19}F]FNT (RI detector, left) and [^{18}F]FNT (Radiation detector, right). *In vitro* stability analysis of [^{18}F]FNT in (C) mouse and (D) human serum at 37 ^{o}C after 60 min (left), and 120 min (right), respectively.

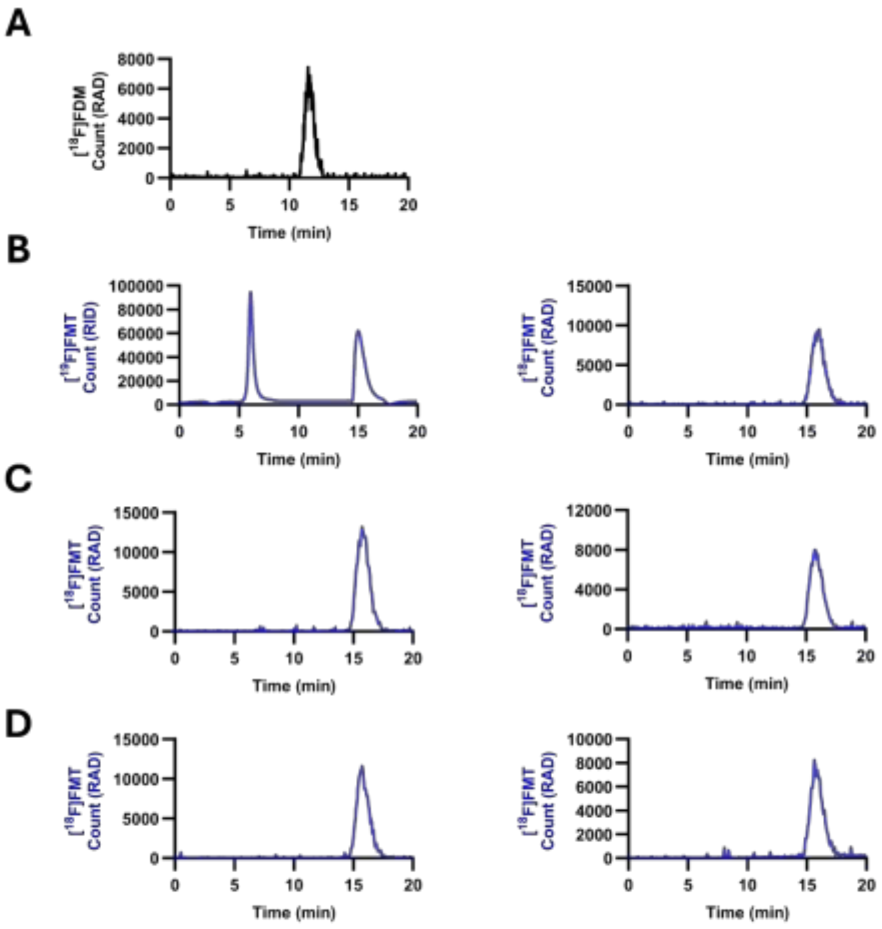

Fig. S3. Analytical-HPLC analysis (YMC-Pack Polyamine II I.D. S-5 µm 250x4.6 mm; 80 % MeCN/Water; 1 mLmin^{-1}) of (A) [^{18}F]FDM, (B) [^{19}F]FMT (RI detector, left) and [^{18}F]FMT (Radiation detector, right). *In vitro* stability analysis of [^{18}F]FMT in (C) mouse and (D) human serum at 37 ^{o}C after 60 min (left), and 120 min (right), respectively.

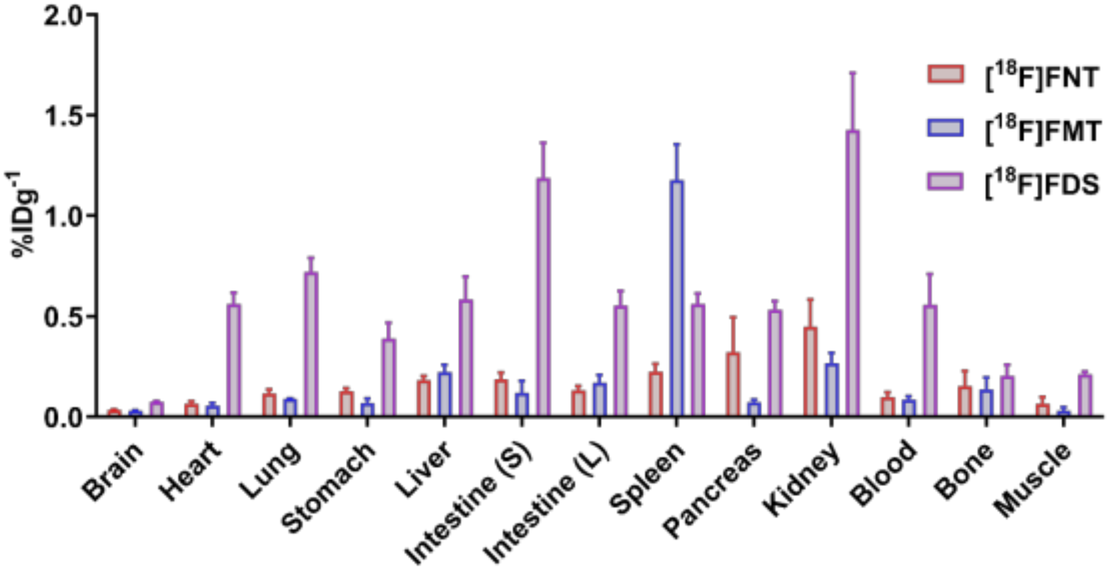

Fig. S4. *Ex vivo* biodistribution of ^{18}F-tracers ([^{18}F]FDS, [^{18}F]FMT and [^{18}F]FNT) in uninfected mice at 100 min post-injection.

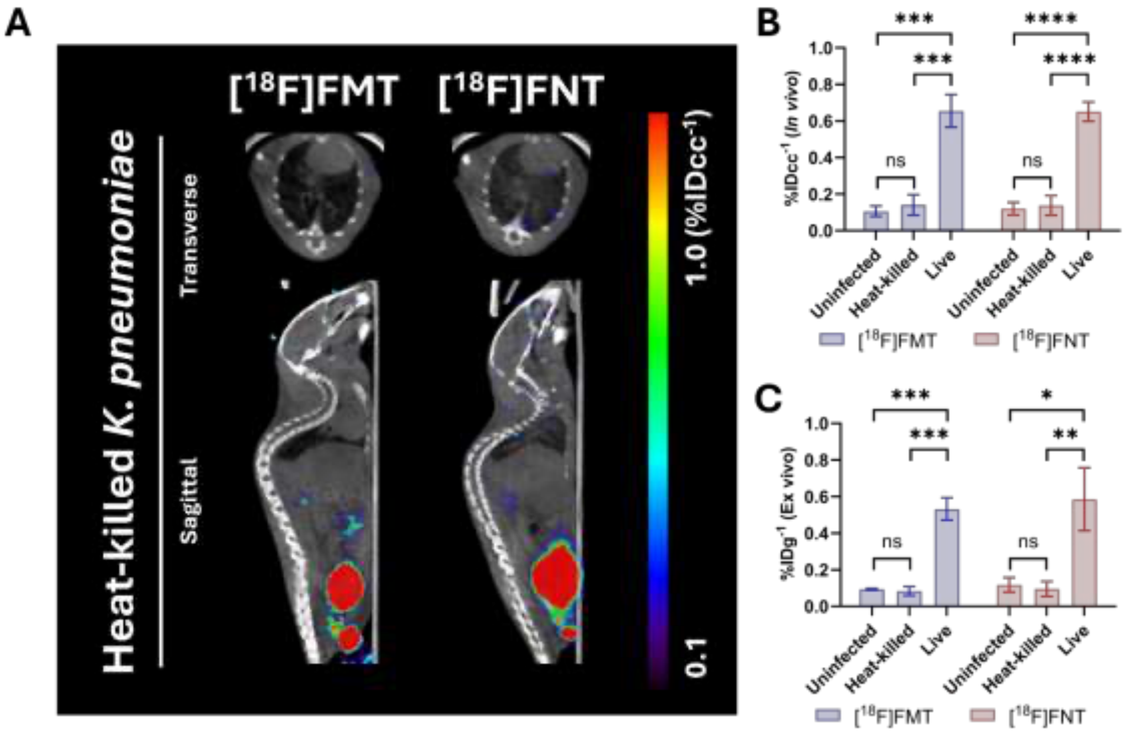

Fig. S5. μPET/CT imaging of ^{18}F-tracers in heat-killed *K. pneumoniae* inoculated mice. (A) Representative μPET/CT images of [^{18}F]FMT and [^{18}F]FNT in heat-killed *K. pneumoniae* inoculated mice (n = 4 for each). (B-C) Comparison of [^{18}F]FMT and [^{18}F]FNT uptake between uninfected, heat-killed and live *K. pneumoniae* inoculated lung *in vivo* (B) and *ex vivo* (C).

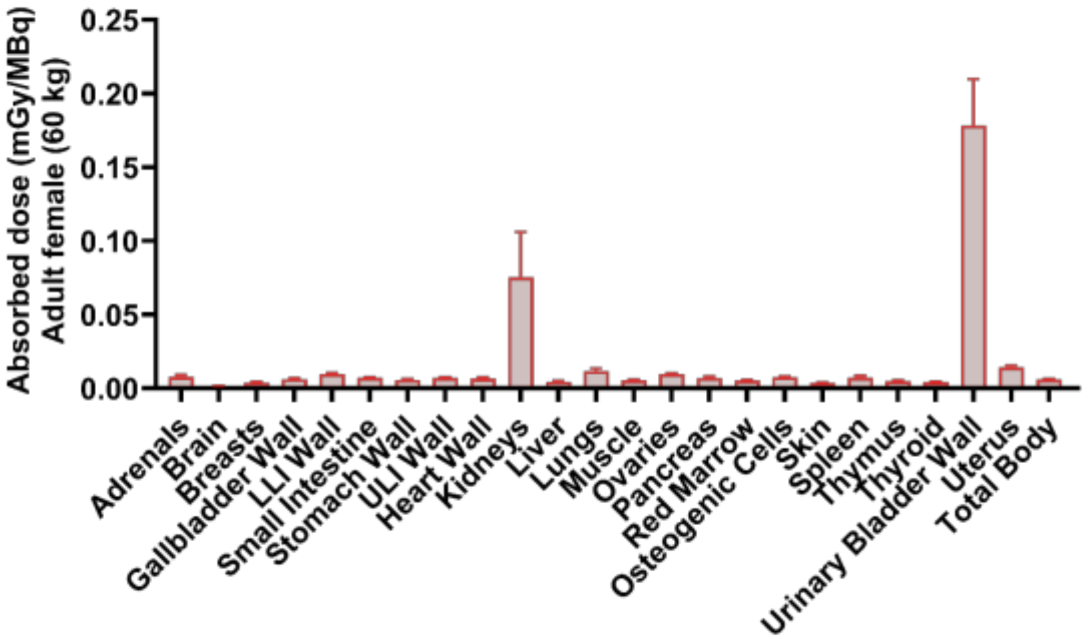

Fig. S6. Estimated absorbed dose (mGy/MBq) extrapolated from murine biodistribution data using ICRP60 (adult female, 60 Kg).

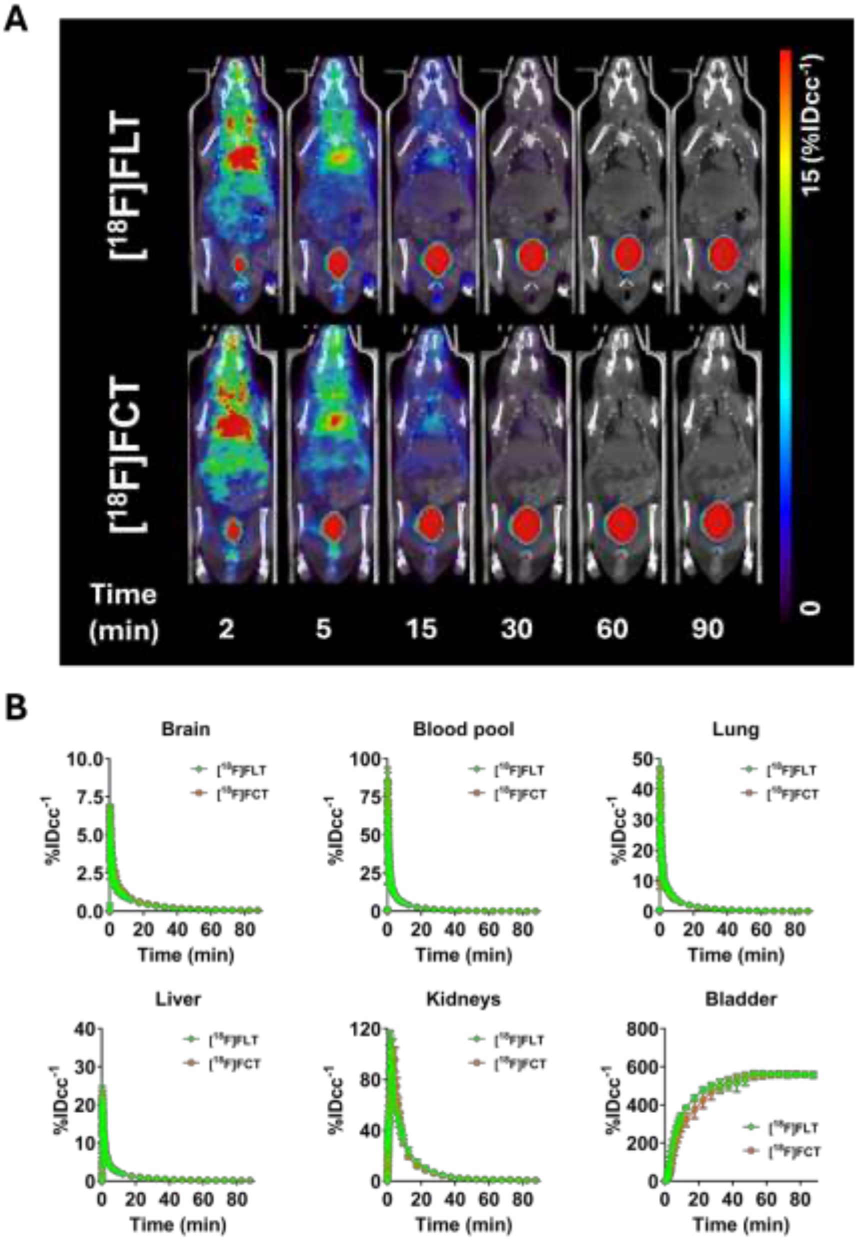

Fig. S7. (A) Representative time-course μPET/CT images of [^{18}F]FLT and [^{18}F]FCT in uninfected mice (n = 4 for each). (B) Time-activity curves (TACs) of blood pool and key organs including brain, lungs, liver, kidneys, and bladder.

.

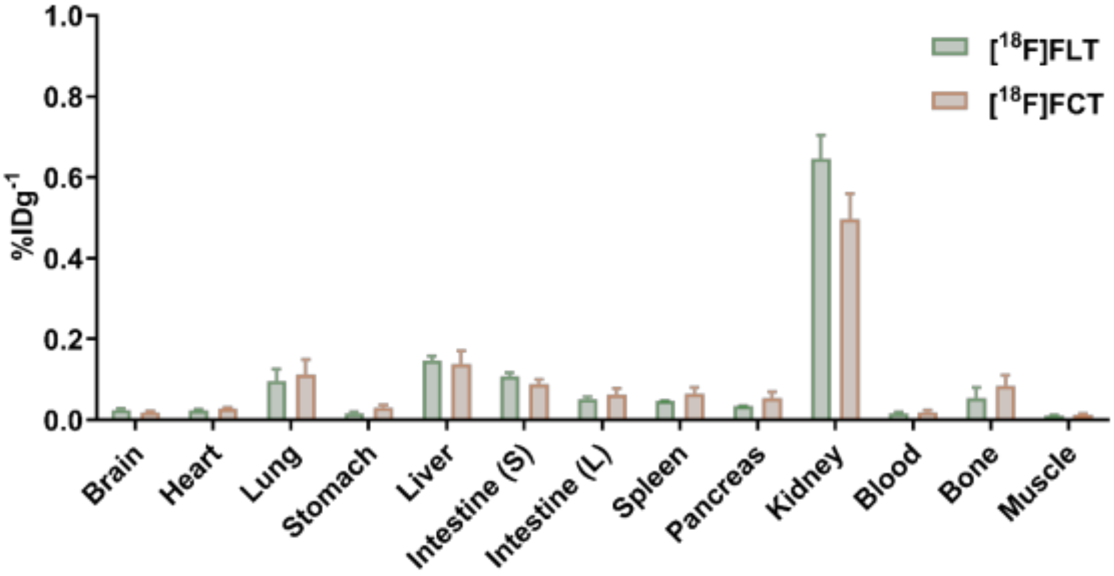

Fig. S8. *Ex vivo* biodistribution of ^{18}F-tracers ([^{18}F]FLT and [^{18}F]FCT) in uninfected mice at 100 min post-injection.

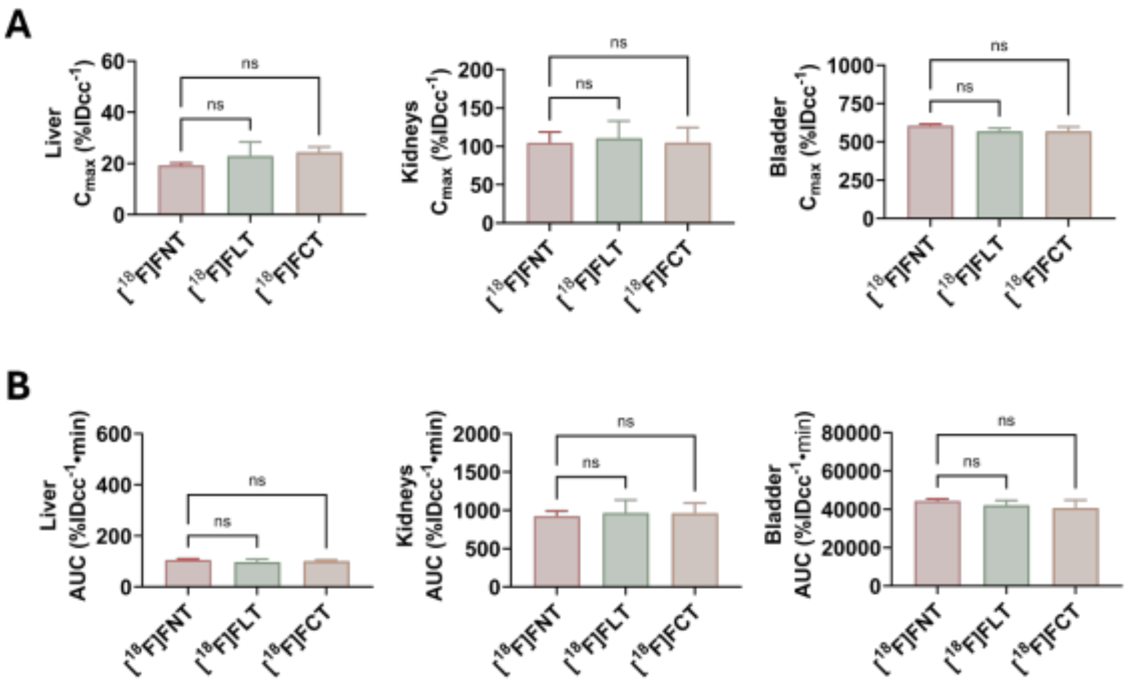

Fig. S9. Comparison of maximum uptake (C_{max}) (A) and area under the curve ($AUC_{0 \rightarrow 90\ min}$) (B) in liver, kidneys and bladder. ns = not significant by one-way ANOVA with Dunnett's multiple comparisons test.

Table S1. Bacterial strains and growth conditions used for this study.

Organism	Strain number	Source	Growth Media
Staphylococcus aureus	*ATCC 29213*	ATCC	Luria Broth
Staphylococcus epidermidis	*ATCC 35984*	ATCC	Luria Broth
Streptococcus agalactiae	*ATCC 13813*	ATCC	Luria Broth
Streptococcus pyogenes	*ATCC 19615*	ATCC	Brain Heart Infusion Broth
Enterococcus faecalis	*ATCC 19433*	ATCC	Luria Broth
Listeria monocytogenes	*ATCC 15313*	ATCC	Luria Broth
Escherichia coli	*ATCC 25922*	ATCC	Luria Broth
Klebsiella pneumoniae	*ATCC 13883*	ATCC	Luria Broth
Enterobacter cloacae	*ATCC 7256*	ATCC	Luria Broth
Acinetobacter baumannii	*ATCC 19606*	ATCC	Luria Broth
S. aureus (MSSA)	-	University of Nebraska Medical Center	Luria Broth
S. aureus (MRSA)	-	University of Nebraska Medical Center	Luria Broth
